# Is Meat Industry Affiliation Associated With Study Conclusion in Nutrition Research? A Meta‐Research Review

**DOI:** 10.1111/obr.70153

**Published:** 2026-05-03

**Authors:** Navid Teimouri, Katherine Sievert, Adam Hannah, Stephanie Meciar, Kerri Gill, Helen Jane Senior, Katherine Cullerton

**Affiliations:** ^1^ School of Public Health University of Queensland Herston QLD Australia; ^2^ Global Centre for Preventive Health and Nutrition, Institute for Health Transformation, School of Health and Social Development Deakin University Geelong VIC Australia; ^3^ School of Political Science and International Studies University of Queensland St. Lucia QLD Australia

**Keywords:** conflicts of interest, health, meat, meta research

## Abstract

**Introduction:**

The meat industry's role in funding and influencing scientific research raises concerns about its impact on evidence used to inform public health policy. Although industry influence on other food and beverage sectors is well‐documented, its effects on studies of meat consumption remain understudied.

**Objective:**

This study aimed to investigate the influence of meat industry involvement on study conclusions of research examining the health impacts of meat consumption.

**Methods:**

A meta‐research review of relevant studies published between 2014 and 2023 was conducted using PubMed and Scopus. Studies investigating the nutritional health impacts of meat consumption were included. Study characteristics, author affiliations, declared funding sources, declared conflicts of interest, and study conclusions were extracted. Association tests were used to assess the relationship between industry ties and study conclusions.

**Results:**

Of 500 included studies, 78 (15.6%) reported industry involvement. Studies with industry ties were 16 times more likely to report favorable conclusions regarding meat consumption (odds ratio [OR] = 16.4, 95% CI: 7.5–35.8), and there was a significant association (*p* < 0.001) between industry involvement and study conclusion.

**Conclusion:**

Meat industry involvement significantly increases the likelihood of favorable study conclusions in nutrition research. These findings underscore the need for caution when interpreting research funded or associated with the meat industry and emphasize the importance of minimizing conflicts of interest in nutrition research.

**Trial Registration:**

Prospero: ID CRD42024526116

## 1 Introduction

Excessive meat production and consumption have become a focal point in public debates, driven by growing concerns over health risks, environmental impacts, and animal welfare implications [1, 2]. Diets high in meat, especially red and processed meats, are associated with high levels of greenhouse gas emissions and an increased risk of noncommunicable diseases (NCD) including cancer, cardiovascular disease, and diabetes [3]. Consumption of red and processed meats is further associated with a greater risk of obesity, which may be attributable in part to their high energy density and saturated fat content [4]. The extent of health risks varies depending on the type of meat, level of processing, and consumption patterns, leading to continuing debates about the nutritional benefits and harms of meat‐rich diets [3]. Beyond NCD risks, meat consumption and production have broader implications, including environmental degradation, antimicrobial resistance, zoonotic diseases, and animal welfare. Despite calls from international expert bodies to reduce consumption of red and processed meat, the cultural and economic importance of meat in many societies has contributed to widespread policy inertia [1, 5]. In this context, meat industry actors may have an interest in producing and disseminating favorable scientific findings, particularly regarding the nutritional health impacts of meat consumption.

Commercial for‐profit organizations (hereafter known as “industry”^1^) are prevalent funders of scientific research, particularly in the health and nutrition field [6, 7, 8, 9]. Since the 1980s, researchers have investigated the phenomenon of “funding effects” or “sponsorship bias” [10], identifying associations between funding sources and research outcomes across various health domains, including drug trials and studies on the health impacts of sugar‐sweetened beverages [11, 12, 13]. Studies have highlighted that industry‐funded research frequently yields favorable conclusions for their products, a trend observed across different industries and forms of support [14, 15, 16]. Odierna et al. [11] identified five key stages in the research process where this influence can happen: setting the research agenda/question, identifying a relevant population, choosing the methods and internal validity, implementation of the study, and publication, which also includes downstream dissemination of results in media or marketing campaigns.

Although multiple factors can influence study conclusions, author affiliation and industry involvement have been identified as an important source of conclusion bias [17, 18, 19]. Previous research has shown that industry ties in research studies can influence conclusions without necessarily influencing results [20, 21], indicating the use of spin in their reporting and the reporting of less reliable conclusions [22, 23]. Conclusions are important because they may be picked up by media publications and can influence how research is interpreted [24, 25]. A 2012 study of spin in press releases [26] identified spin in abstract conclusions as the single strongest predictor for spin in press releases. Press releases are an important source for medical media coverage, emphasizing the importance of conclusions for media coverage [26]. Some readers may also skim read research studies and focus on their conclusions to build their opinions [27]. This creates opportunities for industry to influence public opinion.

Industry's interest in funding nutrition‐related research broadly serves to support sales volumes and underscore the social and political legitimacy of its products and practices [9]. It may do so both by emphasizing the health and nutritional benefits of certain products, or by obfuscating the long‐term health risks and environmental impacts associated with high levels of meat consumption. This research can then be used by the industry to promote their products, influence consumer choices, and/or shape dietary or public health guidelines [28]. Moreover, it can also function as a means to persuade policy‐makers or legitimize policy decisions [29, 30], constituting a key component of the food industry's broader corporate political activity [31]. In short, industry influence has the potential to significantly undermine both the reliability and credibility of scientific research and the quality of policy development, regulatory, and consumer decisions.

However, although detailed studies have examined industry influence on research in several food and beverage sectors [14, 15, 32], the meat industry has been largely unexamined. This represents a critical research gap. Ongoing societal and scientific debates about meat consumption and production, coupled with the substantial financial resources of some meat industry groups, highlight their potential to significantly influence research. Particularly trade and industry associations stand out as important actors in this context [1]. For example, the industry financed Beef and Pork Checkoff^2^ programs in the United States utilize up to a quarter of their annual funding (150 million USD) for research purposes [33, 34]. Similarly, Meat and Livestock Australia invested around 327 million AUD for research, development and marketing purposes in 2023–2024, with some of this allocated specifically into nutrition research with the goal to “inform policy discussion on the role of Australian red meat and contribute to communicating an evidence‐based narrative on its nutritional and health benefits in a sustainable diet” [35]. Hence, this meta‐research review aims to better understand the influence of the meat industry on study conclusions and to determine to what extent, if any, meat industry involvement in nutrition research yields more favorable conclusions for meat products compared to nonindustry‐affiliated research. In line with research on other industries' involvement in science, we hypothesized that studies with ties to the meat industry are more likely to conclude favorably about the benefits of meat consumption.

## 2 Methods

Meta‐research is the study of research itself, including its methods, reporting practices, or incentive structures [36]. One valuable contribution of meta‐research is the identification of biases in research, for example, biases related to study design or publication [37]. As part of this broader field, we conducted a review of studies examining the effect of meat consumption on human health. Our study is registered with PROSPERO (CRD42024526116). This review is described here in accordance with the Preferred Reporting Items for Systematic reviews and Meta‐Analyses (PRISMA) statement [38].

Patients and the public were not involved in any way in this study as it used secondary data from previous studies only.

### 2.1 Sample and Search Strategy

We searched two databases, PubMed and Scopus, in December 2023 to identify relevant publications from 2014 to 2023. The timeframe was chosen due to the significant attention meat production and consumption have gained in the last decade [39, 40, 41] and the higher research transparency standards established in the late 2000s [42], which potentially resulted in more rigorous reporting of conflicts of interest and funding. The search strategy was developed with the support of two public health librarians and combined relevant terms for meat consumption and nutritional health outcomes. The complete search string is available in Data S1. The search strategy originally also contained terms to identify studies that examined meat production and environmental effects. However, due to the large number of articles published on both outcomes, the present review focuses on nutritional health outcomes only.

#### 2.1.1 Inclusion and Exclusion Criteria

We included all peer‐reviewed research articles of various study designs that reported on the nutritional impacts of meat consumption on human health. Only studies published in English were included. Commentaries, perspectives, protocols, editorials, or similar were excluded. Studies investigating dietary patterns or general risk factors of disease unspecified to meat were excluded. A full list of inclusion and exclusion criteria can be found in Data S1.

#### 2.1.2 Study Selection

References were exported into EndNote 20 [43] and then imported into Covidence [44], where duplicates were removed, and all screening and data extraction were conducted. For title and abstract screening and full text screening, 10% of the articles were double screened independently by a second reviewer (KG and HS), and discrepancies were resolved either by discussion or by consulting a third reviewer (KC).

### 2.2 Data Extraction

For all included papers, the data extracted included title, authors, year of publication, study design, type of meat investigated, assessed health outcome/s, author affiliations, declared conflicts of interest, declared funding sources, the role of the funders, the name of the funders, and the affiliation of the authors, and the conclusion. The data extraction template was piloted on 10 studies, and changes were made to improve clarity and comprehensiveness. Data were then extracted by one reviewer (NT) with 10% independently checked by a second reviewer (KG). Discrepancies were resolved either through discussion or by consulting a third reviewer (KC).

### 2.3 Data Analysis

We hypothesized that studies with some degree of meat industry involvement, via either declared conflicts of interest, research funding, or author affiliation, were more likely to report favorable conclusions about the investigated meat product compared to studies with no industry connections. The primary outcome assessed was the study's conclusion, specifically the key findings of the study highlighted by the authors in the concluding paragraphs. If there was no conclusion section available, the final paragraphs from the discussion section were assessed. The classification of a conclusion as favorable, unfavorable, or neutral/mixed was assessed based on criteria outlined in Table 1. Classifications were undertaken by one reviewer (NT) and 10% independently checked by a second reviewer (KG), and discrepancies were resolved through discussion or consultation of a third reviewer (KC). Reviewers classified conclusions before knowing each study's industry affiliations to mitigate any potential influence during assessment.

**TABLE 1:** Conclusion classification.

**Favorable:** No statements are made to cast the product or practice in a negative light, and, at the same time, the conclusions suggest one or more of the following: Beneficial human health effects The absence of expected adverse human health effects Increased nutritional value	**Unfavorable:** No statements are made that cast the product or practice in a positive light and, at the same time, the conclusions suggest one or more of the following: Adverse human health effects The absence of expected beneficial human health effects Decreased nutritional value	**Neutral/mixed:** A statement is made that the study is inconclusive, or Failed to meet criteria for either favorable or unfavorable classification, or The study reports mixed findings for different types of meat, or The study reports mixed findings for different health outcomes.

The critical appraisal score was used as a secondary measure to assess the methodological rigor of studies [45]. Critical appraisals are commonly used in systematic reviews to evaluate the quality of the included evidence [46]. Due to the variety of study designs included in this review, the Mixed Methods Appraisal Tool (MMAT) was deemed the most suitable tool for assessing the quality and rigor of the studies [47]. Assessment was carried out by several reviewers (NT, KD, SM, and MA) with a 10% sample independently double assessed. Discrepancies were resolved either through discussion or by consulting a third reviewer. Depending on how many criteria of the MMAT were met, the studies received a score between 0% and 100%. If no criteria were met, the study received a score of 0%, and if all five criteria were met, the score was 100%. Interventional studies and observational studies were appraised using the MMAT. For systematic reviews, the critical appraisal tool from the Centre for Evidence‐Based Medicine (CEBM) at the University of Oxford was used due to a similar scoring system [48]. Non‐systematic literature reviews, modeling studies, nutritional analyses, and economic analyses were not eligible for appraisal. The spreadsheet used for the appraisal did not include any indication of industry involvement, and reviewers were instructed to assess the full texts without knowledge of such involvement. No studies were excluded or weighted based on appraisal scores. The scores were used exclusively to give a simple estimate of study quality to assess whether this may be the main factor predicting conclusions.

### 2.4 Industry Influence Measures

As there are a range of ways that industry can be involved in research, three different categories of possible industry ties with the study were identified and assessed: (1) author affiliation, (2) declared conflicts of interest, and (3) declared funding sources. Author affiliations were classified into one of three categories. Table 2 provides an overview of all industry influence classifications.

**TABLE 2:** Industry involvement classification.

Category	Definition
*Industry affiliation*	
Affiliation with industry	At least one of the authors is affiliated with the meat industry or industry‐friendly organizations. Example: An author affiliation with a company involved in meat production.
No affiliation with industry	None of the authors are affiliated with the meat industry or industry‐friendly organizations. Example: All authors affiliated with universities with no explicit ties to the meat industry.
Undetermined	The affiliations of the authors were unclear, with no explicit indication of ties to the meat industry or industry‐friendly organizations one way or another. Example: An author affiliation with a consultancy, which cannot clearly be identified as connected to the meat industry or not.
*Conflicts of interest*	
Conflicts of interest	Conflicts of interest regarding to the meat industry are declared.
No conflicts of interest	The authors declared no conflicts of interest.
Undeclared	The research article provides no information about conflicts of interest.
*Funding sources*	
Industry	Declared funding from corporations or organizations with industry association or organizations that represent industry, such as Meat and Livestock Australia
Nonindustry	Declared funding from governmental institutions with no industry associations, universities, philanthropies, nonprofit organizations, and independent foundations
Unknown or undeclared	Insufficient information about the funding sponsors provided, or if financial sponsorship was not declared by the authors.
No funding received	If it was specifically declared that the study received no external funding

*Note:* Classifications were undertaken by one reviewer (NT) and 10% independently checked by a second reviewer (KG). Any discrepancies were resolved through discussion or consultation of a third reviewer (KC).

### 2.5 Statistical Analysis

We reported frequencies and percentages of the extracted study characteristics, assessed conclusions, and critical appraisal scores across the three domains of industry influence and a pooled group of all studies that presented any type of influence. To evaluate the association between the domains of industry influence and study conclusion, a chi‐squared test of independence was used. For author affiliation, the expected frequencies did not meet the necessary values for a chi‐squared test. Hence, Fisher's exact test was used as an alternative [49]. Using logistic regression analysis, we calculated odds ratios for industry influenced studies to have favorable conclusions compared to noninfluenced studies. The logistic regression analysis was extended to adjust for critical appraisal ratings and study design. All statistical analyses were independently conducted by two authors by using SPSS [50] and R software [51].

## 3 Results

A total of 500 articles met the inclusion criteria and were included in the review (see PRISMA Flow diagram, Figure 1). Interrater reliability for the double‐screened subset was moderate to substantial (Cohen's *κ* = 0.56–0.69; proportionate agreement = 84%–86%). Most of the included studies were observational (*n* = 280, 56%), followed by systematic reviews (*n* = 83, 16.6%), literature reviews (*n* = 63, 12.6%), and randomized controlled trials (*n* = 47, 9.4%). The most frequently assessed health outcomes were cancer (*n* = 152, 30.4%), cardiovascular diseases (*n* = 108, 21.6%), and diabetes (*n* = 70, 14%). Other frequently assessed outcomes were mortality, nutritional outcomes, obesity, and general health. A range of studies assessed a variety of health outcomes within the same analysis. The included studies were published across 195 different journals, with industry influenced studies appearing in 44 of these. Notably, 15 out of 78 (19%) of the industry influenced studies were published in *Nutrients*. All extracted data and the associated critical appraisal can be found in Data S2.

**FIGURE 1:**
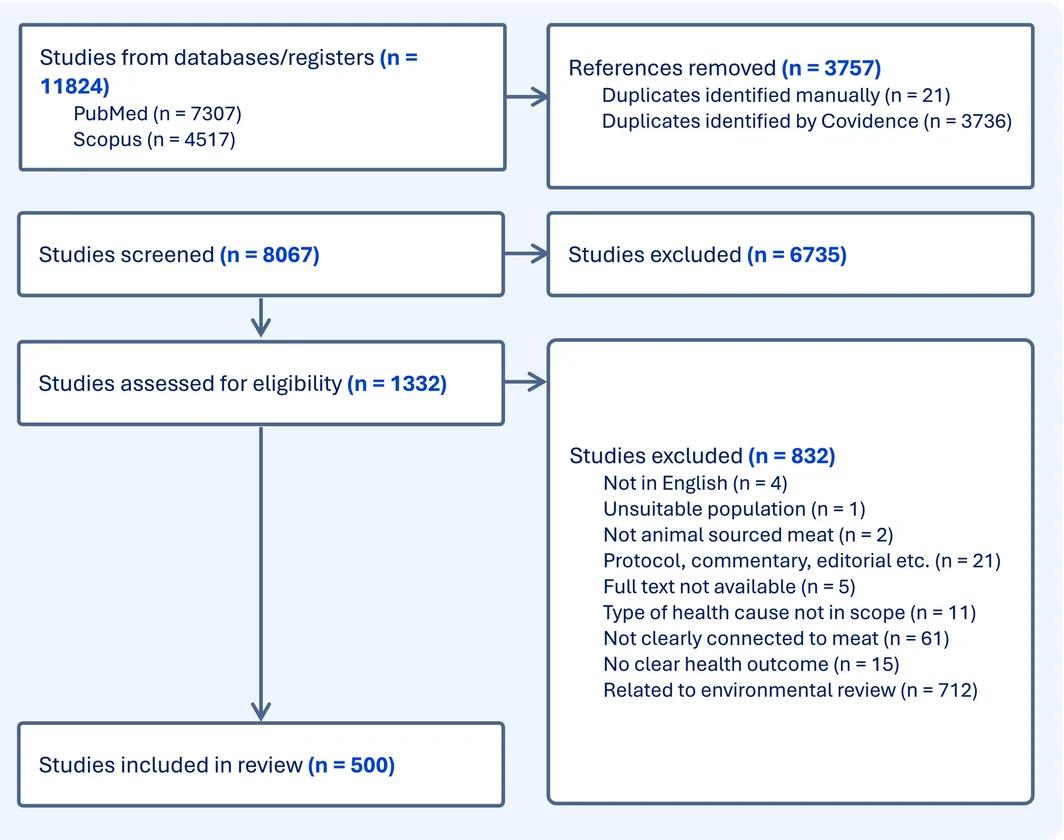
PRISMA flowchart of included articles.

### 3.1 Meat Industry Influence and Conclusion

Around half (*n* = 262, 52%) of all study conclusions reported unfavorable health consequences associated with meat consumption in specific quantities, whereas 133 studies (27%) identified neutral/mixed outcomes, and 105 studies (21%) presented favorable conclusions. As shown in Figure 2, a small proportion (*n* = 78, 15.6%) of the 500 included studies declared any type of meat industry influence, which included authors affiliated with the meat industry, declared conflicts of interest with industry, or industry funding. For an additional 99 studies (20%), it could not be determined whether the author affiliations, conflicts of interest, or funding sources were connected to the meat industry. Author affiliation was the least frequent type of influence, with only 16 studies (3%) indicating an author affiliation with the meat industry. Conflicts of interest were declared in 40 studies (8%), and 63 studies (13%) declared receiving funding from the meat industry for their study. Of the 63 studies that declared funding from the meat industry, only 33 (52%) declared conflicts of interest, and only three (5%) declared an active role of the sponsor in research design or analysis. Figure 2 provides an overview of the declared influences and the number of studies included.

**FIGURE 2:**
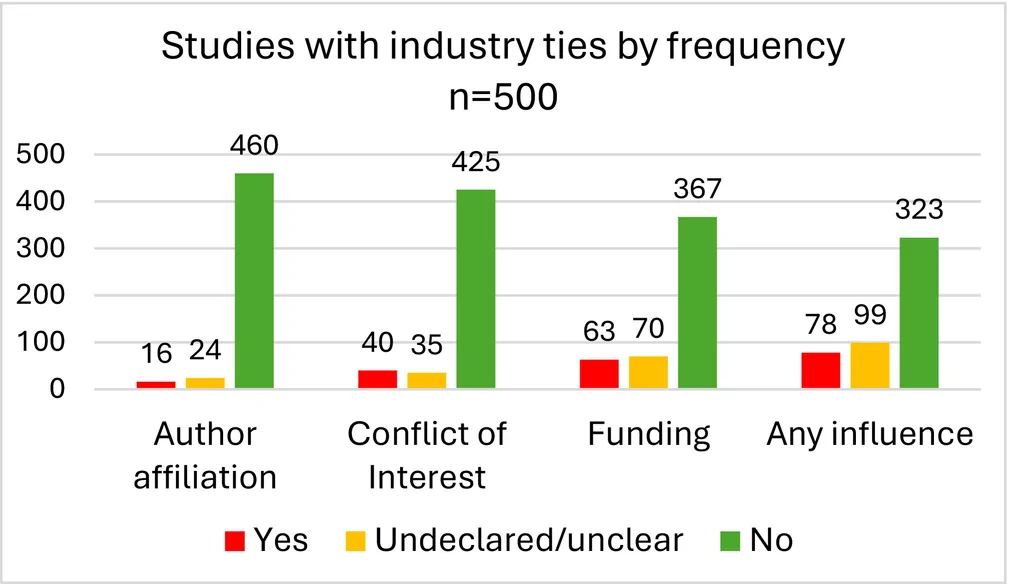
Industry influence on studies by frequency.

The most frequent form of industry tie was meat industry funding of research, primarily through American Checkoff programs such as Beef Checkoff and Pork Checkoff. Across different countries and regions, similar representative associations (e.g., Meat and Livestock Australia, Danish Agriculture and Food Council, UK Agriculture and Horticulture Development Board) were the primary funders of industry‐supported research. Direct collaboration or funding from meat corporations or companies was rare; instead, industry associations were identified as research supporters. For the studies with identified industry ties, most were published by lead authors based in the United States (62%), compared to 14% of nonindustry studies published by US‐based lead authors.

When analyzing the conclusions of the included studies, 75% (*n* = 47) of industry‐funded studies reported favorable nutritional health outcomes associated with meat consumption, whereas only one study concluded an unfavorable association. In comparison, independently funded studies overwhelmingly reported unfavorable (*n* = 225, 61%) or neutral/mixed (*n* = 104, 28%) conclusions, with only 10% (*n* = 38) reporting favorable outcomes in the conclusion. When examining the 16 studies authored by individuals affiliated with the meat industry, all conclusions reported favorable health outcomes related to meat consumption. In contrast, only 17% of studies authored by researchers without industry affiliations provided favorable conclusions. Similar patterns were observed with declared conflicts of interest: 57% of studies disclosing a meat‐related conflict of interest reported favorable conclusions for the meat product, whereas only 5% reported unfavorable conclusions. In contrast, among studies with no declared conflicts of interest with the meat industry, 16% reported favorable conclusions, and 58% unfavorable. Across all forms of industry ties studies were significantly more likely to report favorable conclusions compared to independent studies, with author affiliation with industry providing the strongest association.

Studies with any form of industry ties produced significantly (*p* < 0.001) more favorable conclusions than studies with no meat industry ties. This relationship also held for studies where industry ties were not able to be determined, due to there being no declaration or otherwise a lack of clarity around the status of author–industry relationships. Although these studies did not report conclusions as consistently favorably as those with declared industry ties, their conclusions were still significantly more favorable than studies with no declared funding, affiliation, or conflicts of interest with the meat industry. Table 3 and Figure 3 provide an overview of all modes of meat industry influence and the associated conclusions.

**TABLE 3:** Industry influence on conclusion.

Mode of influence	Conclusion				Association test
	Favorable *n*(%)	Neutral/mixed *n*(%)	Unfavorable *n*(%)	Total	
**Author affiliation**				500	*p* < 0.001
Yes	16 (100)	0	0	16	
Unclear	10 (42)	4 (17)	10 (42)	24	
No	79 (17)	129 (28)	252 (55)	460	
**Conflicts of interest**				500	*p* < 0.001
Yes	23 (57.5)	15 (37.5)	2 (5)	40	
Undeclared	15 (43)	8 (23)	12 (34)	35	
No	67 (16)	110 (26)	248 (58)	425	
**Meat industry funding**				500	*p* < 0.001
Yes	47 (75)	15 (24)	1 (1)	63	
Unclear	20 (29)	14 (20)	36 (51)	70	
No	38 (10)	104 (28)	225 (61)	367	
**Any industry influence**				500	*p* < 0.001
Yes	54 (69)	21 (27)	3 (4)	78	
Unclear	23 (23)	24 (24)	52 (53)	99	
No	28 (9)	88 (27)	207 (64)	323	

**FIGURE 3:**
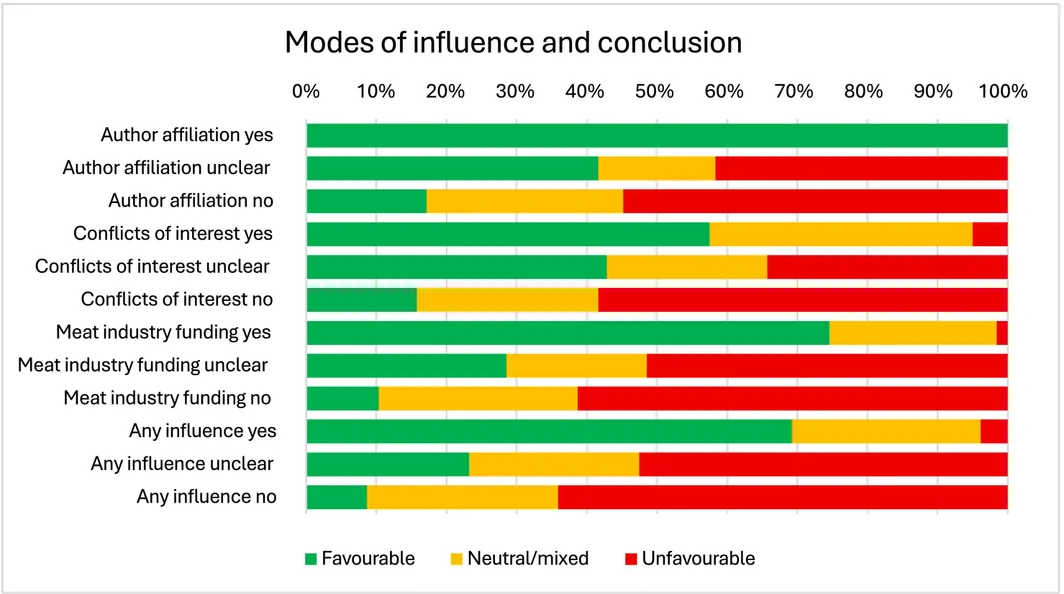
Industry influence on conclusion.

The unadjusted logistic regression analysis also revealed a significant association between meat industry ties and the likelihood of favorable conclusions. Studies with declared industry ties were approximately 23 times more likely (*p* < 0.001, 95% CI: 12.7–43.9) to conclude favorably regarding the meat product. Furthermore, studies with undeclared or undetermined industry associations were also approximately three times more likely (*p* < 0.001, 95% CI: 1.7–5.8) to report favorable conclusions.

### 3.2 Critical Appraisal Rating and Study Conclusion

Of the 500 papers reviewed, 395 were eligible to be appraised using the MMAT or the CEBM systematic review appraisal tool. Studies not eligible included those with study designs not suitable for MMAT appraisal, such as, non‐systematic literature reviews or modeling studies. The average score across all studies was 76.8%, indicating a moderate to high methodological quality. The mean score was significantly lower among experimental studies (52.8%), which often lacked the rigorous reporting of methodology required to achieve higher scores. Systematic reviews scored an average of 79%, whereas observational studies scored 80.3%. Publications with any form of industry ties scored a lower average score (61.3%) compared to those without industry influence (79.6%), indicating lower methodological quality. However, industry studies included significantly more experimental studies, which generally scored lower. To assess whether the favorable conclusions in industry‐tied studies were due to their lower appraisal scores and choice of study design, a logistic regression was adjusted for these factors. In the adjusted model, which included only studies eligible for appraisal (*n* = 391) and accounted for differences in study design and appraisal scores, the odds of industry papers reporting favorable conclusions was 16 times higher than independent papers (*p* < 0.001, 95% CI: 7.5—35.8, Table 4). These results suggest that although study design and appraisal rating may act as confounders in explaining the relationship between industry influence and conclusion, industry ties remain the primary driver.

**TABLE 4:** Results of adjusted logistic regression.

Variable	*p*	(Exp(B)) Odds ratio	95% CI (lower)	95% CI (Upper)
No industry ties	< 0.001			
Industry ties	< 0.001	16.409	7.521	35.801
Unclear	< 0.001	3.359	1.647	6.85
Appraisal Score	0.407	0.999	0.996	1.002
Experimental study	0.128			
Observational study	0.501	0.743	0.313	1.764
Systematic review	0.055	0.379	0.14	1.022
Constant	< 0.001	0.184	—	—

## 4 Discussion

Our review is the first examination of the meat industry's influence on research findings. We identified a strong association between studies with meat industry ties and the reporting of favorable conclusions. This finding was consistent across three modes of industry ties: author affiliation, conflicts of interest, and funding. Association tests and logistic regression supported the hypothesis that industry studies are more likely to report favorable conclusions for the associated meat products. Specifically, studies with meat industry ties were 16 times more likely to report favorable conclusions for meat products than nonindustry‐affiliated studies. These findings support existing evidence that food industry involvement in scientific research often leads to favorable results or conclusions for the industry [14, 15, 24, 32, 52, 53]. Industry can leverage these findings to issue press releases, a key tool for promoting medical research, to attract media attention, or to influence policy and regulation [6, 26].

Although only a small proportion of included studies reported any type of meat industry involvement in the research, this aligns with findings from other reviews on food industry influence in peer‐reviewed research on various food products [32]. While our classification and analysis of conclusions were comparable to previous studies, our findings revealed much stronger associations. Studies with meat industry ties reported highly favorable conclusions and rarely produced unfavorable conclusions. In comparison, an analysis of systematic reviews investigating the effects of sugar‐sweetened beverages by Bes‐Rastrollo et al. [15] found that 83% of industry‐funded papers reported favorable conclusions, while Lesser et al.'s [14] analysis of the beverage industry identified that 63% of studies with industry ties reported favorable conclusions. This similarity suggests that the meat industry is as active as the other food sectors in supporting research that aligns with its interests.

Almost half of the studies that received meat industry funding did not declare a conflict of interest (52%). This suggests that authors may not perceive financial sponsorship as a relevant conflict of interest. Similar analyses of conflict of interest reporting among drug researchers and physicians have also found high numbers of missing and incomplete conflict of interest declarations [54, 55, 56]. Given the results of this and other analyses, there may be limited understanding among researchers of what constitutes a conflict of interest, or a purposeful ignoring of this. To prevent this occurring in the future, authors could use tools like the FoRK guidance and toolkit to identify and assess potential conflicts of interest when collaborating with industry and to transparently declare them [57]. Moreover, a standardized, publicly available, and global conflicts of interest registry has been suggested by researchers previously [58]. This could help researchers, policy‐makers, health professionals, and other stakeholders identify, track, and account for conflicts of interest in medical research [58].

In our analysis, studies with undeclared or undetermined ties to the meat industry were three times more likely to report favorable conclusions for the meat product than those without such ties. These findings are concerning and suggest that our statistical findings of industry involvement are underestimates. We therefore encourage caution when interpreting findings where there is a lack of transparency over funding arrangements or conflicts of interest [55, 59, 60]. Further, our study highlights the importance of transparency guidelines in scientific research and the need for their enforcement through medical and nutrition journals and their editors [61, 62]. Journal editors and reviewers should remain vigilant to potential conflicts of interest and their impact on study conclusions. Our analysis showed that industry‐associated papers were published across a wide range of journals, including high‐impact ones, highlighting that this is not confined to specific journals. Nonetheless, while transparency is a minimal requirement for managing conflicts of interest, it does not necessarily prevent industry influence on research outcomes. Therefore, efforts should focus on actively reducing and eliminating conflicts of interest and strengthening independent sources of research support to prevent biased findings from disproportionately influencing the evidence base [63, 64]. In terms of financial dependence, alternative funding models to minimize funder influence on research outcomes could be strengthened. For example, cross‐sectoral pooled funding may be considered for this purpose [65].

Interestingly, none of the studies with ties to the meat industry were funded by or associated with individual meat companies, including multinational corporations, such as JBS, Cargill, or Tyson Foods. Instead, the connections were typically with industry associations and organizations such as American Beef and Pork Checkoff programs or the Australian Meat and Livestock Association. This contrasts with other food and beverage sectors, where large corporations such as Coca‐Cola, Mars inc., or Nestle have been more directly involved in sponsoring favorable nutrition research [66, 67]. Using representative associations allows large companies to lobby and promote their interests without being seen as directly responsible [68, 69, 70]. This strategy may also be applicable to the research space.

In other sectors, calls for increased transparency and curbs on industry influence in scientific research have seen industry actors respond with proposals for self‐regulation. For example, authors from the Institute for the Advancement of Food and Nutrition Sciences (formerly known as International Life Sciences Institute), an organization funded by industry partners such as Coca‐Cola, McDonalds, and PepsiCo, have published guidelines for protecting the integrity and credibility of the scientific record [71]. These authors argue for prioritizing the science over the conflicts of interest that may influence it. Similarly, some industry‐affiliated authors emphasize that quality appraisal tools should be the sole measure considered when evaluating research papers [72]. We found that while industry studies may differ in methodological quality, this does not sufficiently explain the large differences in their conclusions compared to independent studies. This aligns with other literature; for example, a 2016 analysis of reviews investigating the effect of artificial sweeteners on weight found differences in outcomes independent of other sources of bias [60]. The results suggest that the issue of favorable outcomes in industry‐associated research is not necessarily related to the scientific rigor of these studies [67]. Instead, factors such as choice of study design, selective reporting, or use of framing and interpretation may contribute to the consistently favorable outcomes demonstrated in industry‐funded studies [6, 28, 73]. Therefore, further research is needed to understand why meat‐industry–associated studies tend to reach more favorable conclusions. This may include detailed qualitative analysis of the design and reporting of the individual studies.

The present study has several strengths and limitations. Among its strengths are the use of a comprehensive and systematic search strategy, a large sample size, rigorous classification of industry influences, study conclusions, and appraisal ratings by multiple independent researchers, and robust statistical data. We also included a variety of study designs in contrast to similar reviews that often focus on a single design. However, there are also limitations. The included studies were restricted to English‐language publications, which may have inadvertently omitted relevant studies in other languages from other countries, including some with high meat consumption. Further, we relied solely on declared information; undeclared author affiliations, conflicts of interest, or study funding may have changed the results of the analysis. Our analysis focused only on study conclusions. A deeper examination of methodology and results may identify the mechanisms through which industry ties manifest. Additionally, the assessment of study quality should be interpreted with caution as the appraisal tools used were chosen to give an estimate of study quality and to rule out study quality as the main influence factor and not to weigh or exclude individual studies as part of the analysis. Finally, while we implemented double screening and reviewing at each step of the analysis, this was limited to 10%–15% of the sample due to the high volume of studies included.

## 5 Conclusion

This review adds to the growing body of evidence demonstrating how industry influence can shape scientific research, its outcomes, and subsequent impacts, such as how research could be reported in the media or used to inform government policy. The findings highlight the need for caution interpreting research with industry ties, which should be evaluated within the broader body of evidence. For those writing media stories, developing nutritional guidelines, training nutrition professionals, or creating educational material, we recommend exercising care when using evidence with ties to industry. This includes evaluating the study design, methods, and results rather than relying on its conclusion. In particular, public health experts should be aware that industry affiliation and funding may influence research conclusions. Further, given the number of studies in this review with unclear or undeclared industry ties, and their tendency to report favorable conclusions for the products investigated, we encourage journals and editors to strengthen requirements for transparency declarations and minimize conflicts of interest.

## Author Contributions

Screening was undertaken by N.T., K.G., and H.S., in consultation with K.C. Data extraction was undertaken by N.T. and K.G., in consultation with K.C., K.S., and A.H. Critical appraisal was undertaken by N.T. and S.M. All authors made substantive intellectual contributions to all aspects of the research and writing of this article and approved the final version. K.C. is the guarantor for the work. N.T., K.G., H.S., and S.M. are public health doctoral researchers. K.C. is a public health nutritionist and policy researcher. K.S. is a political economy of food systems researcher. A.H. is a public policy researcher and expert on health care policy. The corresponding author attests that all listed authors meet authorship criteria and no other meeting the criteria have been omitted.

## Funding

There was no funding received for this work. N.T., K.G., S.M., and H.J.S. are recipients of an Australian government research training program scholarship for higher degree by research students. KS. is supported by an Alfred Deakin Postdoctoral Research Fellowship.

## Ethics Statement

This study did not require ethics approval as it was based solely on the analysis of previously published studies and did not involve any human participants, identifiable personal data, or direct interaction with research subjects.

## Conflicts of Interest

All authors have completed the Unified Competing Interest form and declare: no support from any organization for the submitted work; no financial relationships with any organizations that might have an interest in the submitted work in the previous 3 years; no other relationships or activities that could appear to have influenced the submitted work.

## Supporting information


**Data S1:** Supporting information.

## Data Availability

The data that supports the findings of this study are available in the supplementary material of this article.

## Endnotes

^1^
The term meat industry refers to all for‐profit actors that comprise the industrial livestock sector, including production, packaging, preservation, and connected representative organizations [1].

^2^
Checkoff programs are US‐based research and promotion programs for particular agricultural commodities. For example, to fund the Beef Checkoff program, all producers selling cattle or calves are legally required to pay one USD per head to their state's Cattlemen's Beef Board [33].
