# Supplementary material for: Is Meat Industry Affiliation Associated With Study Conclusion in Nutrition Research? A Meta‐Research Review
Source: Obes Rev. 2026 May 3;27(11):e70153. doi: 10.1111/obr.70153 (PMC13655965; doi:10.1111/obr.70153)
Supplement: Supplementary file 1 [file OBR-27-e70153-s001.pdf]

Is meat industry affiliation associated with study conclusion in nutrition research? A meta-research review

Navid Teimouri, Higher Degree by Research Scholar, School of Public Health, University of Queensland, Herston, QLD, Australia, ORCiD: 0000-0002-3340-3688

Katherine Sievert, Alfred Deakin Post-Doctoral Research Fellow, Global Centre for Preventive Health and Nutrition, Institute for Health Transformation, School of Health and Social Development, Deakin University, Geelong, VIC, Australia, ORCiD: 0000-0003-4617-8034

Adam Hannah, Lecturer, School of Political Science and International Studies, University of Queensland, St. Lucia, QLD, Australia, ORCiD: 0000-0002-5063-5755

Stephanie Meciar, Higher Degree by Research Scholar, School of Public Health, University of Queensland, Herston, QLD, Australia, ORCiD: 0009-0002-2589-9043

Kerri Gill, Higher Degree by Research Scholar, School of Public Health, University of Queensland, Herston, QLD, Australia, ORCiD: 0000-0002-7937-9695

Helen Jane Senior, Higher Degree by Research Scholar, School of Public Health, University of Queensland, Herston, QLD, Australia, ORCiD: 0000-0002-1460-7036

Katherine Cullerton, Associate Professor, School of Public Health, University of Queensland, Herston, QLD, Australia, ORCiD: 0000-0001-9517-6380

Correspondence to Navid Teimouri (n.teimouri@uq.edu.au).

Supplementary file 1

Search strategy

Scopus

(((TITLE-ABS ("processed meat*") OR TITLE-ABS ("red meat") OR TITLE-ABS (pork) OR TITLE-ABS (beef) OR TITLE-ABS (lamb) OR TITLE (meat) OR TITLE-ABS ("animal protein")) AND (TITLE-ABS (diet) OR TITLE-ABS (consum*) OR TITLE-ABS (supplement*) OR TITLE-ABS (intake) OR TITLE-ABS (food*)) AND (TITLE-ABS (cancer*) OR TITLE-ABS (mortality) OR TITLE-ABS (nutrition*) OR TITLE-ABS (obesity) OR TITLE-ABS (overweight) OR (TITLE-ABS (diabet*) OR TITLE-ABS ("cardiovascular disease*") OR TITLE-ABS ("health risk factor*") OR TITLE-ABS ("heart disease") OR TITLE-ABS (hypertension) OR TITLE-ABS ("coronary disease") OR TITLE-ABS ("metabolic health") OR TITLE-ABS ("mental health")) AND PUBYEAR > 2013 AND PUBYEAR < 2024))) OR (((TITLE-ABS (meat OR cattle OR pig OR pork OR beef OR lamb OR sheep OR animal* OR livestock)) W/1 (TITLE-ABS (farming OR production))) AND ((TITLE-ABS ("greenhouse gas emission*") OR TITLE-ABS ("carbon footprint") OR TITLE-ABS (methane) OR TITLE-ABS (sequestration) OR TITLE-ABS ("carbon dioxide") OR TITLE-ABS ("CO2 emission*"))) AND PUBYEAR > 2013 AND PUBYEAR < 2024) AND NOT TITLE-ABS ("animal feed" OR aquacultur* OR fisher* OR phyto* OR "in vitro" OR biogas OR toxi* OR "animal health"

OR "meat quality" OR "cultured meat*" OR immuno* OR microbiom*) AND NOT (TITLE (dairy OR milk OR acid*)) AND (LIMIT-TO (DOCTYPE , "ar") OR LIMIT-TO (DOCTYPE , "re")) AND (LIMIT-TO (LANGUAGE , "English"))

PubMed

((((("red meat*"[tiab] OR "processed meat*"[tiab] OR "pork"[tiab] OR "beef"[tiab] OR "lamb"[tiab] OR "meat"[Title] OR "animal protein"[tiab]) AND (Diet[tiab] OR Consum*[tiab] OR supplement*[tiab] OR intake[tiab] OR food*[tiab]) AND ("cancer*"[tiab] OR mortality[tiab] OR nutrition*[tiab] OR obesity[tiab] OR overweight[tiab] OR diabet*[tiab] OR "cardiovascular disease*"[tiab] OR "health risk factor*"[tiab] OR "heart disease"[tiab] OR hypertension [tiab] OR coronary disease[tiab] OR "metabolic health"[tiab] OR mental health[tiab])))) OR (((meat[tiab] cattle[tiab] OR pig[tiab] OR pork[tiab] OR Beef[tiab] OR lamb[tiab] OR animal*[tiab] or livestock[tiab])) AND (farming[tiab] OR production[tiab]) AND ("greenhouse gas emission*"[tiab] OR "carbon footprint"[tiab] OR "methane"[tiab] OR sequestration[tiab] OR "carbon dioxide"[tiab] OR "CO2 emission*"[tiab])) AND (2014:2023[pdat])) NOT ("Animal Feed"[Mesh] OR "animal feed"[tiab] OR aquacultur*[tiab] OR fisher*[tiab] OR phyto*[tiab] OR "in vitro"[tiab] OR biogas*[tiab] OR toxi*[tiab] OR "animal health"[tiab] OR "meat quality"[tiab] OR "cultured meat*"[tiab] OR immune*[tiab] OR microbiome*[tiab]) AND (2014:2023[pdat])) NOT (dairy[Title] OR milk[Title] OR acid*[Title] AND (2014:2023[pdat]))

Data inclusion and exclusion criteria in detail

Inclusion:

- Association between (commonly farmed) meat consumption and human health (or associated risk factors)
- Meat products or compounds typically found in meat (e.g. iron, nitrite, protein) and their health effects
- Comparisons between meat/non-meat products and their health outcomes
- English language
- Peer reviewed published journal articles
- 2014-present
- Electronically available

Exclusion:

- Studies investigating risk factors for certain health conditions more broadly, unspecified to meat
- Studies investigating dietary patterns/guidelines more broadly, unspecified to meat
- Studies investigating health effects through meat contamination (heavy metals, bacteria etc.)
- Animal health and veterinary studies
- Meat sciences
- Social sciences like consumer perceptions or policy recommendations/analyses
- Economic analyses of food/meat production/consumption
- Meat substitute products or cultured meat products unless directly comparing health effects to meat
- Immunology or biochemistry
- Food security
- Fish or seafood
- Meat quality
- Other animal products like eggs or dairy
- Non-English language

Title	Authors	Year	Journal type	Journal name	Country of lead	Country investigated	Study design	Type of meat	Assessed health outcome	Conclusion	Author affiliation	If industry affiliated:	Conflicts of interest	Funding	If industry sponsoring: Which	If industry sponsoring: How	Conclusion	Conclusion merged	Author affiliation	Meat related	Meat related	Meat industry	Any meat industry	Critical
Meat consumption providing a surplus energy in modern diet contributes to obesity prevalence: An ecological analysis	W. You and M. Henneberg	2016	Nutrition	BMC Nutrition	Australia	Global	Observational study	All meat	Obesity	Negative	Non-industry		No	No funding received			Negative	Negative/neutral	Non-industry	No	No	No	No	100
Unprocessed red meat intakes are associated with increased inflammation, triglycerides and HDL cholesterol in past smokers	T. Wu, S. Sonoda and H. Liu	2020	Nutrition	Nutrition and Dietetics	United States	United States	Observational study	Red meat	Other: Inflammation and blood lipid	Neutral/mixed	Non-industry		No	Non-industry			Neutral/mixed	Negative/neutral	Non-industry	No	No	No	No	100
Effects of Adding Lean Red Meat to a U.S.-Style Healthy Vegetarian Dietary Pattern on Gut Microbiota and Cardiovascular Risk	Wang, Y.; Lindemann, S. R.; Cross, T. W. L.; Tang, M.; Clark, C. M.; Campbell, W. W.	2021	Nutrition	Journal of Nutrition	United States	United States	Experimental study	Red meat	Cardiovascular disease; Other: Gut	Positive	Non-industry		Yes	Industry	Pork Checkoff, North Dakota Beef	Not involved in either	Positive	Positive	Non-industry	No	Yes	Yes	Yes	0
Effect of replacing animal protein with plant protein on glycemic control in diabetes: A systematic review and meta-analysis of	Viguiliouk, E.; Stewart, S. E.; Jayalath, V. H.; Ng, A. P.; Mirrahimi, A.; de Souza, R. J.;	2015	Nutrition	Nutrients	Canada	Global	Systematic review	Animal protein	Diabetes	Neutral/mixed	Non-industry		Yes	Non-industry			Neutral/mixed	Negative/neutral	Non-industry	No	Yes	No	Yes	80
Meat and Colorectal Cancer: Associations and Issues	Trudo, S. P.; Gallaher, D. D.	2015	Nutrition	Current Nutrition Reports	United States	Not applicable (e.g. no	Review	All meat	Cancer	Neutral/mixed	Non-industry		No	Undeclared			Neutral/mixed	Negative/neutral	Non-industry	No	No	Undeclared	Unclear/Undeclared	
Red meat consumption and breast cancer risk in premenopausal women: A systematic review and meta-analysis	A. Rezaianzadeh, M. Ghorbani, S. Rezaeian and A. Kassani	2018	Disease specific	Middle East Journal of Cancer	Iran	Global	Systematic review	Red meat	Cancer	Negative	Non-industry		No	Non-industry			Negative	Negative/neutral	Non-industry	No	No	No	No	60
Nutritional score of meat products at retail in Serbia	M. RaÅ¡eta, I. B. LaziÄ‡, B. MrdoviÄ‡, B. Zsolt, S. Mila, M. GrubiÄ‡ and J.	2020	Other: Meat technology	Meat Technology	Serbia	Serbia	Other: Nutritional	All meat	Nutritional outcomes	Negative	Unclear/Undeclar		No	Non-industry			Negative	Negative/neutral	Unclear/Undeclared	Unclear/Undeclar	No	No	Unclear/Undeclared	
Pork Consumption and Its Relationship to Human Nutrition and Health: A Scoping Review	L. P. Penkert, R. Li, J. Huang, A. Gurcan, M. Chung and T. C. Wallace	2021	Other: Meat and Muscle	Meat and Muscle Biology	United States	Not applicable (e.g. no	Review	Pork	Cardiovascular disease; Nutritional	Positive	Industry	Think Healthy Group	Yes	Industry	National Pork Board	Not involved in either	Positive	Positive	Industry	Yes	Yes	Yes	Yes	
A pilot study to determine the effects of lean beef consumption on markers of metabolic syndrome	K. L. Olson, M. B. Russell, K. Raml, G. D. Dizra and K. K. Kattelmann	2016	Nutrition	Functional Foods in Health and	United States	United States	Experimental study	Beef	Cardiovascular disease	Positive	Non-industry		No	Industry	South Dakota Beef Industry Council,	Undeclared	Positive	Positive	Non-industry	No	No	Yes	Yes	20
Red Meat and Health: Getting to the Heart of the Matter	L. E. O'Connor and W. W. Campbell	2017	Nutrition	Nutrition Today	United States	Not applicable (e.g. no	Review	Red meat	General health	Neutral/mixed	Non-industry		Yes	Undeclared			Neutral/mixed	Negative/neutral	Non-industry	No	Yes	Undeclared	Yes	
Vitamin D Biofortification of Pork May Offer a Food-Based Strategy to Increase Vitamin D Intakes in the UK Population	H. R. Neill, C. I. R. Gill, E. J. McDonald, W. C. McRoberts and L. K. Pourshahidi	2021	Nutrition	Frontiers in Nutrition	UK	UK	Observational study	Pork	Other: Vitamin D levels	Positive	Industry	Devenish Nutrition Ltd	No	Industry	Devenish Nutrition Ltd.	Involved in analysis	Positive	Positive	Industry	Yes	No	Yes	Yes	
Dietary Animal Protein vs. Plant Protein Sources in Relation to the Risk of Biliary Stone: A Case-Control Study	K. Naseri, S. Saadati, F. Pashayee-Khamene, A. Hekmatdoost, H. Asadzadeh-	2023	Nutrition	Journal of Nutrition and Food Security	Iran	Iran	Observational study	Animal protein	Other: Biliary stone	Negative	Non-industry		No	Non-industry			Negative	Negative/neutral	Non-industry	No	No	No	No	
Effect of dry-cured ham rich in bioactive peptides on cardiovascular health: A randomized controlled trial	L. Montoro-GarcÃa, M. P. Zafrilla-Rentero, F. M. CelÃ¡n-de Haro, J.	2017	Other: Food	Journal of Functional Foods	Spain	Spain	Experimental study	Pork	Cardiovascular disease	Positive	Non-industry		No	Non-industry			Positive	Positive	Non-industry	No	No	No	No	60
Dietary intake of red meat, processed meat, and poultry and risk of colorectal cancer and all-cause mortality in the context of	H. Meijborn, S. P. MÃ¸ller, L. C. Thygesen and A. Biltoft-Jensen	2020	Nutrition	Nutrients	Denmark	Denmark	Observational study	Poultry; Red meat;	Mortality; Cancer	Neutral/mixed	Non-industry		No	Non-industry			Neutral/mixed	Negative/neutral	Non-industry	No	No	No	No	60
Coming to terms: Meat's role in a healthful diet	S. H. McNeill, K. E. Belk, W. W. Campbell and C. L. Gifford	2017	Other: Animal Frontiers	Animal Frontiers	United States	Not applicable (e.g. no	Review	All meat	General health	Positive	Industry	National Cattlemen's	Undeclared	Undeclared			Positive	Positive	Industry	Yes	Undeclared	Undeclared	Yes	
Animal and plant proteins: Which effects on cardiometabolic health?	F. Mariotti	2019	Nutrition	Medecine des Maladies Metaboliques	France	Not applicable (e.g. no	Review	Animal protein	Cardiovascular disease; Other: Metabolic	Negative	Non-industry		No	No funding received			Negative	Negative/neutral	Non-industry	No	No	No	No	
Effect of plant protein on blood lipids: A systematic review and meta-analysis of randomized controlled trials	S. S. Li, S. B. Mejia, L. Lytvyn, S. E. Stewart, E. Viguiliouk, V. Ha, et al.	2017	Medicine broadly	Journal of the American Heart	Canada	Global	Systematic review	Animal protein	Other: Cholesterol	Negative	Non-industry		Yes	Non-industry			Negative	Negative/neutral	Non-industry	No	Yes	No	No	60
Associations between red meat intake and biomarkers of inflammation and glucose metabolism in women	S. H. Ley, Q. Sun, W. C. Willett, A. H. Eliassen, K. Wu, A. Pan, et al.	2014	Nutrition	American Journal of Clinical Nutrition	United States	United States	Observational study	Red meat	Other: Inflammation and glucose metabolism	Negative	Non-industry		Yes	Non-industry			Negative	Negative/neutral	Non-industry	No	No	No	No	80
Adopting a Mediterranean-style eating pattern with low, but not moderate, unprocessed, lean red meat intake reduces fasting	S. Krishnan, L. E. O'Connor, Y. Wang, E. R. Gertz, W. W. Campbell and B. J. Bennett	2022	Nutrition	British Journal of Nutrition	United States	United States	Experimental study	Beef	Obesity; Other: Fasting biomarkers	Neutral/mixed	Non-industry		Yes	Industry	Beef Checkoff, Pork Checkoff	Undeclared	Neutral/mixed	Negative/neutral	Non-industry	No	Yes	Yes	Yes	80
Partial replacement of red and processed meat with legumes: A modelling study of the impact on nutrient intakes and nutrient	N. E. Kaartinen, H. Tapanainen, M. Maukonen, E. PÃ¤ivÃ¤rinta, L. M. Valsta, S. T.	2022	Nutrition	Public Health Nutrition	Finland	Not applicable (e.g. no	Other: Modeling study	Red meat; Processed	Nutritional outcomes	Negative	Non-industry		No	Non-industry			Negative	Negative/neutral	Non-industry	No	No	No	No	
The Importance of Poultry Meat in Medicine: A Review	S. A. Jilo and L. A. Hasan	2022	Other: Poultry research	Journal of World's Poultry Research	Ethiopia	Not applicable (e.g. no	Review	Poultry	General health	Positive	Unclear/Undeclar		No	No funding received			Positive	Positive	Unclear/Undeclared	Unclear/Undeclar	No	No	Unclear/Undeclared	
The link between vegetarian diet and depression might be explained by depression among meat-reducers	C. J. Hopwood	2022	Other: Food	Food Quality and Preference	Switzerland	North America	Observational study	All meat	Other: Mental Health	Neutral/mixed	Non-industry		No	Undeclared			Neutral/mixed	Negative/neutral	Non-industry	No	No	Undeclared	Unclear/Undeclared	40
Are Plant-Based Meat Alternative Products Healthier Than the Animal Meats They Mimic?	L. J. Harnack, M. M. Reese and A. J. Johnson	2022	Nutrition	Nutrition Today	United States	Not applicable (e.g. no	Review	All meat; Other: Meat	Nutritional outcomes	Neutral/mixed	Non-industry		No	Non-industry			Neutral/mixed	Negative/neutral	Non-industry	No	No	No	No	
Dietary intake of animal and plant proteins and risk of all cause and cause-specific mortality: The Epic-Italy cohort	L. Fontana, S. Sieri, F. Ricceri, C. Agnoli, V. Pala, G. Masala, et al.	2021	Nutrition	Nutrition and Healthy Aging	Australia	Italy	Observational study	Animal protein	Mortality	Negative	Non-industry		No	Non-industry			Negative	Negative/neutral	Non-industry	No	No	No	No	60
The role of meat in iron nutrition of vulnerable groups of the UK population	S. Fairweather-Tait	2023	Other: Animal Science	Frontiers in Animal Science	UK	Not applicable (e.g. no	Review	All meat	Nutritional outcomes	Neutral/mixed	Non-industry		No	Non-industry			Neutral/mixed	Negative/neutral	Non-industry	No	No	No	No	
Red meat consumption and type 2 diabetes mellitus risk: A systematic review of the evidence	E. Derbyshire and C. Ruxton	2015	Nutrition	Nutrition and Food Science	UK	Global	Systematic review	Red meat	Diabetes	Positive	Industry	Nutritional Insight	No	Industry	Meat Advisory Panel, Meat	Not involved in either	Positive	Positive	Industry	Yes	No	Yes	Yes	40
Protein-enriched diet, with the use of lean red meat, combined with progressive resistance training enhances lean tissue mass and	Daly, R. M.; O'Connell, S. L.; Mundell, N. L.; Grimes, C. A.; Dunstan, D. W.; Nowson,	2014	Nutrition	American Journal of Clinical Nutrition	Australia	Australia	Experimental study	Red meat	Obesity; Cardiovascular disease; Muscle	Positive	Non-industry		No	Industry	Meat and Livestock Australia	Not involved in either	Positive	Positive	Non-industry	No	No	Yes	Yes	60
The association between protein consumption from animal and plant sources with psychological distress in older people in the	N. M. D'Cunha, A. Kouris, S. Tyrovolas, C. Chrysohoou, L. Rallidis, E.	2020	Nutrition	Nutrition and Healthy Aging	Australia	Greece	Observational study	Animal protein	Other: Mental health	Negative	Non-industry		Undeclared	Non-industry			Negative	Negative/neutral	Non-industry	No	Undeclared	No	Unclear/Undeclared	100
The Effect of Dietary Protein Intake on the Risk of Gestational Diabetes	Chen, L.; Du, S.; Song, H.; Chen, J.; Lv, C.; Li, C.	2022	Other: Food	Journal of Food Quality	China	Global	Systematic review	Animal protein	Diabetes	Negative	Non-industry		No	Undeclared			Negative	Negative/neutral	Non-industry	No	No	Undeclared	Unclear/Undeclared	100
Nutritional quality and physiological effects of halal meat: A pilot study in non-Muslim consumers	Carrasco, C.; PÃ©rez-Palacios, T.; Luengo, L. M.; GÃ³mez, G.; Fajardo, M.; GÃ³mez,	2022	Other: Food	Food Bioscience	Spain	Spain	Experimental study	Other: Halal meat	Nutritional outcomes; General health	Positive	Non-industry		No	Industry	Golden Worldwide Trade S.L.	Not involved in either	Positive	Positive	Non-industry	No	Yes	Yes	Yes	0
Evidence on the potential effects of halal meat on sleep/wake cycles and mood state profile: A pilot study	Carrasco, C.; GonzÃ¡lez-Mateos, A.; GÃ³mez, M. A.; RodrÃguez, A. B.	2022	Other: Food	International Food Research Journal	Spain	Spain	Experimental study	Other: Halal meat	Other: Sleep	Positive	Non-industry		No	Industry	Golden Worldwide Trade S.L.	Not involved in either	Positive	Positive	Non-industry	No	Yes	Yes	Yes	20
An overview of the nutritional value of beef and lamb meat from South America	Cabrera, M. C.; Saadoun, A.	2014	Other: Meat Science	Meat Science	Uruguay	Not applicable (e.g. no	Review	Beef; Lamb	Nutritional outcomes	Positive	Non-industry		Undeclared	Undeclared			Positive	Positive	Non-industry	No	Undeclared	Undeclared	Unclear/Undeclared	
Review: Nutrient density and nutritional value of meat products and non-meat foods high in protein	B. Bohrer	2017	Other: Food Science	Trends in Food Science and Technology	Canada	Not applicable (e.g. no	Review	All meat	Nutritional outcomes	Neutral/mixed	Non-industry		Undeclared	No funding received			Neutral/mixed	Negative/neutral	Non-industry	No	Undeclared	No	Unclear/Undeclared	
Processed meat intake is unfavorably and fish intake favorably associated with semen quality indicators among men attending a	M. C. Afeiche, A. J. Gaskins, P. L. Williams, T. L. Toth, D. L. Wright, C. Tanrikut, et al.	2014	Nutrition	Journal of Nutrition	United States	United States	Observational study	Processed meat	Other: Semen quality	Negative	Non-industry		No	Non-industry			Negative	Negative/neutral	Non-industry	No	No	No	No	100
Association of Processed Meats and Alcohol Consumption with Renal Cell Carcinoma: A Worldwide Population-Based Study	I. Ziouziou, A. M. Touzani, L. Lahlou, S. F. Shariat, F. Sanguedolce, Y. Neuzillet, et al.	2021	Nutrition	Nutr Cancer	Morocco	Global	Observational study	Processed meat	Cancer	Negative	Non-industry		No	Undeclared			Negative	Negative/neutral	Non-industry	No	No	Undeclared	Unclear/Undeclared	40
Association of meat consumption and changes with all-cause mortality in hypertensive patients during 11.4-year follow-up:	P. Zhuang, J. Jiao, F. Wu, L. Mao and Y. Zhang	2021	Nutrition	Clin Nutr	China	China	Observational study	All meat	Cardiovascular disease; Mortality	Negative	Non-industry		No	Non-industry			Negative	Negative/neutral	Non-industry	No	No	No	No	80
Animal-based food choice and associations with long-term weight maintenance and metabolic health after a large and rapid	R. Zhu, M. Fogelholm, E. Jalo, S. D. Poppitt, M. P. Silvestre, G. MÃ¸ller, et al.	2022	Nutrition	Clin Nutr	Denmark	Global	Observational study	Animal protein	Obesity; Other: Metabolic health	Neutral/mixed	Non-industry		Yes	Industry	The Cambridge Weight Plan, The	Not involved in either	Neutral/mixed	Negative/neutral	Non-industry	No	Yes	Yes	Yes	60
Meat consumption is associated with esophageal cancer risk in a meat- and cancer-histological-type dependent manner	H. C. Zhu, X. Yang, L. P. Xu, L. J. Zhao, G. Z. Tao, C. Zhang, et al.	2014	Disease specific	Dig Dis Sci	China	Global	Systematic review	All meat	Cancer	Negative	Non-industry		No	Non-industry			Negative	Negative/neutral	Non-industry	No	No	No	No	100
Associations of Processed Meat, Unprocessed Red Meat, Poultry, or Fish Intake With Incident Cardiovascular Disease and All-Cause	V. W. Zhong, L. Van Horn, P. Greenland, M. R. Carnethon, H. Ning, J. T. Wilkins, et al.	2020	Medicine broadly	JAMA Intern Med	United States	United States	Observational study	Poultry; Red meat;	Cardiovascular disease; Mortality	Negative	Non-industry		Yes	Non-industry			Negative	Negative/neutral	Non-industry	No	No	No	No	80
Protein foods from animal sources, incident cardiovascular disease and all-cause mortality: a substitution analysis	V. W. Zhong, N. B. Allen, P. Greenland, M. R. Carnethon, H. Ning, J. T. Wilkins, et al.	2021	Other: Epidemiology	Int J Epidemiol	United States	United States	Observational study	Animal protein	Cardiovascular disease; Mortality	Negative	Non-industry		Yes	Non-industry			Negative	Negative/neutral	Non-industry	No	No	No	No	80
Association of changes in red meat consumption with total and cause specific mortality among US women and men: two	Y. Zheng, Y. Li, A. Satija, A. Pan, M. Sotos-Prieto, E. Rimm, et al.	2019	Medicine broadly	BMJ	China	United States	Observational study	Red meat	Mortality	Negative	Non-industry		No	Non-industry			Negative	Negative/neutral	Non-industry	No	No	No	No	100
The Isocaloric Substitution of Plant-Based and Animal-Based Protein in Relation to Aging-Related Health Outcomes: A Systematic	J. Zheng, T. Zhu, G. Yang, L. Zhao, F. Li, Y. M. Park, et al.	2022	Nutrition	Nutrients	China	China	Systematic review	Animal protein	Diabetes	Negative	Non-industry		No	Non-industry			Negative	Negative/neutral	Non-industry	No	No	No	No	80
Red and processed meat consumption and gastric cancer risk: a systematic review and meta-analysis	Z. Zhao, Z. Yin and Q. Zhao	2017	Disease specific	Oncotarget	China	Global	Systematic review	Red meat; Processed	Cancer	Negative	Non-industry		No	Non-industry			Negative	Negative/neutral	Non-industry	No	No	No	No	40
Association Between Consumption of Red and Processed Meat and Pancreatic Cancer Risk: A Systematic Review and Meta-analysis	Z. Zhao, Z. Yin, Z. Pu and Q. Zhao	2017	Other: Gastroenterology	Clin Gastroenterol Hepatol	China	Global	Systematic review	Red meat; Processed	Cancer	Neutral/mixed	Non-industry		No	Undeclared			Neutral/mixed	Negative/neutral	Non-industry	No	No	Undeclared	Unclear/Undeclared	60
Red and processed meat consumption and esophageal cancer risk: a systematic review and meta-analysis	Z. Zhao, F. Wang, D. Chen, C. Zhang	2020	Disease specific	Clin Transl Oncol	China	China	Systematic review	Red meat; Processed	Cancer	Negative	Non-industry		No	Non-industry			Negative	Negative/neutral	Non-industry	No	No	No	No	60
Red and processed meat consumption and colorectal cancer risk: a systematic review and meta-analysis	Z. Zhao, Z. Feng, Z. Yin, J. Shuang, B. Bai, P. Yu, et al.	2017	Disease specific	Oncotarget	China	Global	Systematic review	Red meat; Processed	Cancer	Negative	Non-industry		No	Non-industry			Negative	Negative/neutral	Non-industry	No	No	No	No	80
Dietary protein intake and risk of type 2 diabetes: a dose-response meta-analysis of prospective studies	L. G. Zhao, Q. L. Zhang, X. L. Liu, H. Wu, J. L. Zheng and Y. B. Xiang	2019	Nutrition	European Journal of Nutrition	China	Global	Meta analysis	Animal protein	Diabetes	Negative	Non-industry		No	Non-industry			Negative	Negative/neutral	Non-industry	No	No	No	No	100
Increased risk of esophageal squamous cell carcinoma associated with frequent and long-term consumption of salted meat and	L. Zhang, Y. C. Li, J. P. Wu, Y. J. Zhao, R. B. Wang, M. Jiang and Q. K. Song	2019	Medicine broadly	J Int Med Res	China	China	Observational study	Processed meat	Cancer	Negative	Non-industry		No	Non-industry			Negative	Negative/neutral	Non-industry	No	No	No	No	80
Red/processed meat consumption and non-cancer-related outcomes in humans: umbrella review	X. Zhang, S. Liang, X. Chen, J. Yang, Y. Zhou, L. Du and K. Li	2023	Nutrition	Br J Nutr	China	Global	Systematic review	Red meat; Processed	Cardiovascular disease; Mortality; Diabetes;	Negative	Non-industry		No	Non-industry			Negative	Negative/neutral	Non-industry	No	No	No	No	60
Processed and Unprocessed Red Meat Consumption and Risk for Type 2 Diabetes Mellitus: An Updated Meta-Analysis of Cohort	R. Zhang, J. Fu, J. B. Moore, L. Stoner and R. Li	2021	Other: Environmental	Int J Environ Res Public Health	China	Global	Meta analysis	Red meat; Processed	Diabetes	Negative	Non-industry		No	Non-industry			Negative	Negative/neutral	Non-industry	No	No	No	No	80
Association of red and processed meat consumption with cardiovascular morbidity and mortality in participants with and without	Zhang, J.; Hayden, K.; Jackson, R.; Schutte, R.	2020	Nutrition	Clin Nutr	UK	UK	Observational study	Red meat; Processed	Obesity; Cardiovascular disease; Mortality	Negative	Non-industry		No	Non-industry			Negative	Negative/neutral	Non-industry	No	No	No	No	80
Meat consumption and risk of incident dementia: cohort study of 493,888 UK Biobank participants	Zhang, H.; Greenwood, D. C.; Risch, H. A.; Bunce, D.; Hardie, L. J.; Cade, J. E.	2021	Nutrition	American Journal of Clinical Nutrition	UK	UK	Observational study	All meat	Other: Dementia	Negative	Non-industry		Yes	Non-industry			Negative	Negative/neutral	Non-industry	No	No	No	No	100
Effect of Lower Versus Higher Red Meat Intake on Cardiometabolic and Cancer Outcomes: A Systematic Review of Randomized	Zeraatkar, D.; Johnston, B. C.; Bartoszko, J.; Cheung, K.; Bala, M. M.; Valli, C.;	2019	Medicine broadly	Ann Intern Med	Canada	Global	Systematic review	Red meat	Cardiovascular disease; Cancer	Neutral/mixed	Non-industry		Yes	Non-industry			Neutral/mixed	Negative/neutral	Non-industry	No	Yes	No	Yes	60
Red and Processed Meat Consumption and Risk for All-Cause Mortality and Cardiometabolic Outcomes: A Systematic Review and	Zeraatkar, D.; Han, M. A.; Guyatt, G. H.; Vernooij, R. W. M.; El Dib, R.; Cheung, K.;	2019	Medicine broadly	Ann Intern Med	Canada	Global	Systematic review	Red meat; Processed	Cardiovascular disease; Mortality	Neutral/mixed	Non-industry		Yes	Non-industry	Conflict of interest statement:		Neutral/mixed	Negative/neutral	Non-industry	No	Yes	No	Yes	80
High red and processed meat consumption is associated with non-alcoholic fatty liver disease and insulin resistance	Zelber-Sagi, S.; Ivancovsky-Wajcman, D.; Fliss Isakov, N.; Webb, M.; Orenstein, D.;	2018	Disease specific	J Hepatol	Israel	Israel	Observational study	Red meat; Processed	Diabetes; Other: Liver disease	Negative	Non-industry		No	Non-industry			Negative	Negative/neutral	Non-industry	No	No	No	No	80
Processed meat, red meat, white meat, and digestive tract cancers: A two-sample Mendelian randomization study	Z. Yun, M. Nan, X. Li, Z. Liu, J. Xu, X. Du, et al.	2023	Nutrition	Front Nutr	China	UK; Finland	Observational study	Poultry; Red meat;	Cancer	Negative	Non-industry		No	Non-industry			Negative	Negative/neutral	Non-industry	No	No	No	No	100
Dose-Response Meta-Analysis on Risk of Diabetes in Relation to Red and Processed Meat Consumption - Asian Populations, 2006-	Yu, H.; Zhang, J.; Xie, J.; Li, X.; Yang, Y.; Zhou, F.; Liu, M.; Zhang, Z.; Shan,	2021	Medicine broadly	China CDC Wkly	China	Asia	Meta analysis	Red meat; Processed	Diabetes	Negative	Non-industry		No	Non-industry			Negative	Negative/neutral	Non-industry	No	No	No	No	100
Total Meat Intake is Associated with Life Expectancy: A Cross-Sectional Data Analysis of 175 Contemporary Populations	You, W.; Henneberg, R.; Saniotis, A.; Ge, Y.; Henneberg, M.	2022	Medicine broadly	Int J Gen Med	Australia	Global	Observational study	All meat	Other: Life Expectancy	Positive	Non-industry		No	Undeclared			Positive	Positive	Non-industry	No	No	Undeclared	Unclear/Undeclared	80
Prostate Cancer Incidence is Correlated to Total Meat Intakeâ€" a Cross-National Ecologic Analysis of 172 Countries	You, W.; Henneberg, M.	2018	Disease specific	Asian Pac J Cancer Prev	Australia	Global	Observational study	All meat	Cancer	Negative	Non-industry		No	Non-industry			Negative	Negative/neutral	Non-industry	No	No	No	No	80
Total meat (flesh) supply may be a significant risk factor for cardiovascular diseases worldwide	You, W.; Feng, S.; Donnelly, F.	2023	Nutrition	Food Sci Nutr	Australia	Global	Observational study	All meat	Cardiovascular disease	Negative	Non-industry		No	Non-industry			Negative	Negative/neutral	Non-industry	No	No	No	No	80
Higher Animal Protein Intake During the Second Trimester of Pregnancy Is Associated With Risk of GDM	Yong, H. Y.; Mohd Shariff, Z.; Mohd Yusof, B. N.; Rejali, Z.; Tee, Y. Y. S.; Bindels, J.;	2021	Nutrition	Front Nutr	Malaysia	Malaysia	Observational study	Animal protein	Diabetes	Negative	Industry	Danone, Nutricia	Yes	Industry	Danone Dumex	Involved in design and	Negative	Negative/neutral	Industry	No	No	No	No	80
A Prospective Analysis of Red and Processed Meat Intake in Relation to Colorectal Cancer in the Black Women's Health Study	Fiannakou, I.; Barber, L. E.; Li, S.; Adams-Campbell, L. L.; Palmer, J. R.; Rosenberg,	2022	Nutrition	J Nutr	United States	United States	Observational study	Red meat; Processed	Cancer	Negative	Non-industry		No	Non-industry			Negative	Negative/neutral	Non-industry	No	No	No	No	100
Red and processed meat intake and risk for cutaneous melanoma in white women and men: Two prospective cohort studies	H. Yen, W. Q. Li, A. Dhana, T. Li, A. Qureshi and E. Cho	2018	Other: Dermatology	J Am Acad Dermatol	United States	United States	Observational study	Red meat; Processed	Cancer	Positive	Non-industry		No	Non-industry			Positive	Positive	Non-industry	No	No	No	No	60
Dietary protein intake and subsequent risk of type 2 diabetes: a dose-response meta-analysis of prospective cohort studies	Ye, J.; Yu, Q.; Mai, W.; Liang, P.; Liu, X.; Wang, Y.	2019	Disease specific	Acta Diabetol	China	Global	Meta analysis	Animal protein	Diabetes	Negative	Non-industry		No	Undeclared			Negative	Negative/neutral	Non-industry	No	No	Undeclared	Unclear/Undeclared	100
Meat and fish intake and type 2 diabetes: Dose-response meta-analysis of prospective cohort studies	Yang, X.; Li, Y.; Wang, C.; Mao, Z.; Zhou, W.; Zhang, L.; Fan, M.; Cui, S.; Li, L.	2020	Disease specific	Diabetes Metab	China	Global	Meta analysis	All meat	Diabetes	Negative	Non-industry		No	Non-industry			Negative	Negative/neutral	Non-industry	No	No	No	No	100
Global burden of ischemic heart disease associated with high red and processed meat consumption: an analysis of 204 countries	Yan, D.; Liu, K.; Li, F.; Shi, D.; Wei, L.; Zhang, J.; Su, X.; Wang, Z.	2023	Medicine broadly	BMC Public Health	China	Global	Observational study	Red meat; Processed	Cardiovascular disease	Negative	Non-industry		No	No funding received			Negative	Negative/neutral	Non-industry	No	No	No	No	80
Red and processed meat consumption and the risk of lung cancer: a dose-response meta-analysis of 33 published studies	Xue, X. J.; Gao, Q.; Qiao, J. H.; Zhang, J.; Xu, C. P.; Liu, J.	2014	Medicine broadly	Int J Clin Exp Med	China	Global	Meta analysis	Red meat; Processed	Cancer	Negative	Non-industry		No	Undeclared			Negative	Negative/neutral	Non-industry	No	No	Undeclared	Unclear/Undeclared	80
Processed Meat Intake and Bladder Cancer Risk in the Prostate, Lung, Colorectal, and Ovarian (PLCO) Cohort	Xu, X.	2019	Disease specific	Cancer Epidemiol Biomarkers Prev	China	China	Observational study	Processed meat	Cancer	Negative	Non-industry		No	Non-industry			Negative	Negative/neutral	Non-industry	No	No	No	No	100
Meat consumption and risk of oral cavity and oropharynx cancer: a meta-analysis of observational studies	Xu, J.; Yang, X. X.; Wu, Y. G.; Li, X. Y.; Bai, B.	2014	Other: Science	PLoS One	China	Global	Meta analysis	All meat	Cancer; Other: Dental health	Neutral/mixed	Non-industry		No	Non-industry			Neutral/mixed	Negative/neutral	Non-industry	No	No	No	No	100
Dietary Protein Patterns during Pregnancy Are Associated with Risk of Gestational Diabetes Mellitus in Chinese Pregnant Women	Wu, W.; Tang, N.; Zeng, J.; Jing, J.; Cai, L.	2022	Nutrition	Nutrients	China	China	Observational study	Animal protein	Diabetes	Negative	Non-industry		No	Non-industry			Negative	Negative/neutral	Non-industry	No	No	No	No	80
Association of Processed Meat Intake with Hypertension Risk in Hemodialysis Patients: A Cross-Sectional Study	Wu, P. Y.; Yang, S. H.; Wong, T. C.; Chen, T. W.; Chen, H. H.; Chen, T. H.; Chen, Y. T.	2015	Medicine broadly	PLoS One	Taiwan	Taiwan	Observational study	Processed meat	Cardiovascular disease	Negative	Non-industry		No	Non-industry			Negative	Negative/neutral	Non-industry	No	No	No	No	100
The relationship between processed meat, red meat, and risk of types of cancer: A Mendelian randomization study	K. Wu, L. Liu, T. Shu, A. Li, D. Xia and X. Sun	2022	Nutrition	Front Nutr	China	UK	Observational study	Red meat; Processed	Cancer	Neutral/mixed	Non-industry		No	Non-industry			Neutral/mixed	Negative/neutral	Non-industry	No	No	No	No	100
Dietary Protein Sources and Incidence of Breast Cancer: A Dose-Response Meta-Analysis of Prospective Studies	Wu, J.; Zeng, R.; Huang, J.; Li, X.; Zhang, J.; Ho, J. C.; Zheng, Y.	2016	Nutrition	Nutrients	China	Global	Meta analysis	Animal protein	Cancer	Negative	Non-industry		No	Non-industry			Negative	Negative/neutral	Non-industry	No	No	No	No	100
Potential health hazards of eating red meat	Wolk, A.	2016	Medicine broadly	J Intern Med	Sweden	Not applicable (e.g. no	Review	All meat	General health	Negative	Non-industry		Yes	Non-industry			Negative	Negative/neutral	Non-industry	No	No	No	No	
Meat consumption and gastric cancer risk: the Japan Public Health Center-based Prospective Study	Wilunda, C.; Yamaji, T.; Iwasaki, M.; Inoue, M.; Tsugane, S.; Sawada, N.	2022	Nutrition	American Journal of Clinical Nutrition	Japan	Japan	Observational study	All meat	Cancer	Positive	Non-industry		No	Non-industry			Positive	Positive	Non-industry	No	No	No	No	100
Meat, Fish, Poultry, and Egg Intake at Diagnosis and Risk of Prostate Cancer Progression	Wilson, K. M.; Mucci, L. A.; Drake, B. F.; Preston, M. A.; Stampfer, M. J.;	2016	Disease specific	Cancer Prev Res (Phila)	United States	United States	Observational study	Poultry; Red meat;	Cancer	Negative	Non-industry		Yes	Non-industry			Negative	Negative/neutral	Non-industry	No	Yes	No	Yes	60
Potential effects of reduced red meat compared with increased fiber intake on glucose metabolism and liver fat content: a	C. Willmann, M. Heni, K. Linder, R. Wagner, N. Stefan, J. Machann, et al.	2019	Nutrition	American Journal of Clinical Nutrition	Germany	Germany	Experimental study	Red meat	Diabetes	Neutral/mixed	Non-industry		No	Non-industry			Neutral/mixed	Negative/neutral	Non-industry	No	No	No	No	40
Red meat consumption is associated with an increased overall cancer risk: a prospective cohort study in Korea	Wie, G. A.; Cho, Y. A.; Kang, H. H.; Ryu, K. A.; Yoo, M. K.; Kim, Y. A.; Jung, K. W.;	2014	Nutrition	Br J Nutr	South Korea	South Korea	Observational study	Red meat	Cancer	Negative	Non-industry		No	Non-industry			Negative	Negative/neutral	Non-industry	No	No	No	No	80
Pre-diagnosis meat intake and cooking method and ovarian cancer survival: results from the Ovarian Cancer Follow-Up Study	Y. F. Wei, M. L. Sun, Z. Y. Wen, F. H. Liu, Y. S. Liu, S. Yan, et al.	2022	Other: Food	Food Funct	China	China	Observational study	All meat	Cancer	Negative	Non-industry		No	Non-industry			Negative	Negative/neutral	Non-industry	No	No	No	No	80
A metabolomic study of red and processed meat intake and acylcarnitine concentrations in human urine and blood	Wedekind, R.; Kiss, A.; Keski-Rahkonen, P.; Viallon, V.; Rothwell, J. A.; Cross, A. J.;	2020	Nutrition	American Journal of Clinical Nutrition	France	France	Experimental study	Pork; Red meat;	Cardiovascular disease	Negative	Non-industry		No	Non-industry			Negative	Negative/neutral	Non-industry	No	No	No	No	40
Pre-diagnostic meat and fibre intakes in relation to colorectal cancer survival in the European Prospective Investigation into Cancer	Ward, H. A.; Norat, T.; Overvad, K.; Dahm, C. C.; Bueno-de-Mesquita, H. B.; Jenab,	2016	Nutrition	Br J Nutr	UK	Europe	Observational study	All meat	Cancer	Neutral/mixed	Non-industry		No	Non-industry			Neutral/mixed	Negative/neutral	Non-industry	No	No	No	No	80
Meat and haem iron intake in relation to glioma in the European Prospective Investigation into Cancer and Nutrition study	Ward, H. A.; Gayle, A.; Jakszyn, P.; Merritt, M.; Melin, B.; Freisling, H.; Weiderpass,	2018	Disease specific	Eur J Cancer Prev	UK	Europe	Observational study	All meat	Cancer	Positive	Non-industry		No	Non-industry			Positive	Positive	Non-industry	No	No	No	No	80
Fatty and lean red meat consumption in China: differential association with Chinese abdominal obesity	Wang, Z.; Zhang, B.; Zhai, F.; Wang, H.; Zhang, J.; Du, W.; Su, C.; Zhang, J.; Jiang, H.;	2014	Nutrition	Nutr Metab Cardiovasc Dis	United States	China	Observational study	Red meat	Obesity	Negative	Non-industry		No	Non-industry			Negative	Negative/neutral	Non-industry	No	No	No	No	100
Moderate Intake of Lean Red Meat was Associated with Lower Risk of Elevated Blood Pressure in Chinese Women: Results from	Wang, Z.; Huang, Q.; Wang, L.; Jiang, H.; Wang, Y.; Wang, H.; Zhang, J.; Zhai, F.;	2015	Nutrition	Nutrients	China	China	Observational study	Red meat	Cardiovascular disease	Positive	Non-industry		No	Non-industry			Positive	Positive	Non-industry	No	No	No	No	100
Red and Processed Meat, Poultry, Fish, and Egg Intakes and Cause-Specific and All-Cause Mortality among Men with	Y. Wang, E. J. Jacobs, R. A. Shah, V. L. Stevens, T. Gansler and M. L. McCullough	2020	Medicine broadly	Cancer Epidemiol Biomarkers Prev	United States	United States	Observational study	Poultry; Red meat;	Mortality; Cancer	Negative	Non-industry		No	Non-industry			Negative	Negative/neutral	Non-industry	No	No	No	No	100
Plant- and Animal-Based Protein-Rich Foods and Cardiovascular Health	Wang, Y.; Hill, E. R.; Campbell, W. W.; O'Connor, L. E.	2022	Disease specific	Current Atherosclerosis Reports	United States	Not applicable (e.g. no	Review	Animal protein	Cardiovascular disease	Positive	Non-industry		Yes	Undeclared			Positive	Positive	Non-industry	No	Yes	Undeclared	Yes	
Red and processed meat consumption and mortality: dose-response meta-analysis of prospective cohort studies	Wang, X.; Lin, X.; Ouyang, Y. Y.; Liu, J.; Zhao, G.; Pan, A.; Hu, F. B.	2016	Nutrition	Public Health Nutrition	China	Global	Meta analysis	Red meat; Processed	Mortality	Negative	Non-industry		No	Non-industry			Negative	Negative/neutral	Non-industry	No	No	No	No	100
Red meat consumption and all-cause and cardiovascular mortality: results from the UK Biobank study	Wang, M.; Ma, H.; Song, Q.; Zhou, T.; Hu, Y.; Heianza, Y.; et al.	2022	Nutrition	European Journal of Nutrition	China	UK	Observational study	Red meat	Cardiovascular disease; Mortality	Negative	Non-industry		No	Non-industry			Negative	Negative/neutral	Non-industry	No	No	No	No	80
Plasma Metabolite Profiles of Red Meat, Poultry, and Fish Consumption, and Their Associations with Colorectal Cancer Risk	Wang, F.; Chandler, P. D.; Zeleznik, O. A.; Wu, K.; Wu, Y.; Yin, K.; Song, R.; Avila-	2022	Nutrition	Nutrients	United States	United States	Observational study	Red meat; Processed	Cancer	Negative	Non-industry		Yes	Non-industry			Negative	Negative/neutral	Non-industry	No	No	No	No	100
A Mediterranean Diet with Fresh, Lean Pork Improves Processing Speed and Mood: Cognitive Findings from the MedPork	Wade, A. T.; Davis, C. R.; Dyer, K. A.; Hodgson, J. M.; Woodman, R. J.; Keage, H. A.	2019	Nutrition	Nutrients	Australia	Australia	Experimental study	Pork	Other: Cognitive skills	Positive	Non-industry		No	Industry	Pork Cooperative Research Centre	Not involved in either	Positive	Positive	Non-industry	No	No	Yes	Yes	80
Meat consumption and colorectal cancer risk in Japan: The Takayama study	Wada, K.; Oba, S.; Tsuji, M.; Tamura, T.; Konishi, K.; Goto, Y.; Mizuta, F.; Koda, S.;	2017	Disease specific	Cancer Sci	Japan	Japan	Observational study	All meat	Cancer	Negative	Non-industry		No	Non-industry			Negative	Negative/neutral	Non-industry	No	No	No	No	80
Intake of different types of red meat, poultry, and fish and incident colorectal cancer in women and men: results from the MalmÃ¶	Vulcan, A.; Manjer, J.; Ericson, U.; Ohlsson, B.	2017	Nutrition	Food & Nutrition Research	Sweden	Sweden	Observational study	Poultry; Red meat;	Cancer	Neutral/mixed	Non-industry		No	Non-industry			Neutral/mixed	Negative/neutral	Non-industry	No	No	No	No	60
Animal Protein Intake Is Associated with Obesity Remission After Roux-en-Y Gastric Bypass: an Isocaloric Replacement Analysis	Vilela, D. L. S.; Silva, A.; Pinto, S. L.; Bressan, J.	2023	Disease specific	Obes Surg	Brazil	Brazil	Observational study	White meat; Animal	Obesity	Positive	Non-industry		No	Non-industry			Positive	Positive	Non-industry	No	No	No	No	40
Approximately Half of Total Protein Intake by Adults Must be Animal-Based to Meet Nonprotein, Nutrient-Based	F. Vieux, D. Remond, J. L. Peyraud and N. Darmon	2022	Nutrition	J Nutr	France	France	Observational study	Animal protein	Nutritional outcomes	Positive	Industry	MS-Nutrition,	No	Industry	French National Interprofessional	Not involved in either	Positive	Positive	Industry	Yes	No	Yes	Yes	
Patterns of Red and Processed Meat Consumption and Risk for Cardiometabolic and Cancer Outcomes: A Systematic Review and	Vernooij, R. W. M.; Zeraatkar, D.; Han, M. A.; El Dib, R.; Zworth, M.; Milio, K.; Sit,	2019	Medicine broadly	Ann Intern Med	Netherlands	Global	Systematic review	Red meat; Processed	Cardiovascular disease; Cancer	Neutral/mixed	Non-industry		Yes	No funding received			Neutral/mixed	Negative/neutral	Non-industry	No	Yes	No	Yes	60
The influence of processed meat consumption on chronic obstructive pulmonary disease	Varraso, R.; Camargo, C. A., Jr.	2015	Medicine broadly	Expert Rev Respir Med	France	Not applicable (e.g. no	Review	Processed meat	Other: Chronic Obstructive Pulmonary	Neutral/mixed	Non-industry		No	Non-industry			Neutral/mixed	Negative/neutral	Non-industry	No	No	No	No	
Partly replacing meat protein with soy protein alters insulin resistance and blood lipids in postmenopausal women with abdominal	M. van Nielen, E. J. Feskens, E. J. Rietman, E. Siebelink and M. Mensink	2014	Nutrition	J Nutr	Netherlands	Netherlands	Experimental study	All meat	Cardiovascular disease	Negative	Non-industry		No	Industry	Alpro Foundation	Undeclared	Negative	Negative/neutral	Non-industry	No	No	No	No	80
Dietary protein intake and incidence of type 2 diabetes in Europe: the EPIC-InterAct Case-Cohort Study	Van Nielen, M.; Feskens, E. J. M.; Mensink, M.; Sluijs, I.; Molina, E.; Amiano, P.;	2014	Disease specific	Diabetes Care	Netherlands	Europe	Observational study	Animal protein	Diabetes	Negative	Unclear/Undeclar		Yes	Non-industry			Negative	Negative/neutral	Unclear/Undeclared	Unclear/Undeclar	No	No	Unclear/Undeclared	100
Association Between Unprocessed Red Meat and Processed Meat With Risk of Recurrence and Mortality in Patients With Stage III	van Blarigan, E. L.; Ou, F. S.; Bainter, T. M.; Fuchs, C. S.; Niedzwiecki, D.; Zhang, S.;	2022	Medicine broadly	JAMA Netw Open	United States	United States	Observational study	Red meat; Processed	Mortality; Cancer	Positive	Unclear/Undeclar		Yes	Non-industry			Positive	Positive	Unclear/Undeclared	Unclear/Undeclar	No	No	Unclear/Undeclared	100
Association between red meat consumption and colon cancer: A systematic review of experimental results	Turner, N. D.; Lloyd, S. K.	2017	Medicine broadly	Exp Biol Med (Maywood)	United States	Global	Systematic review	Red meat	Cancer	Positive	Non-industry		No	Industry	National Cattlemen's Beef	Undeclared	Positive	Positive	Non-industry	No	No	Yes	Yes	
Health Implications of Beef Intramuscular Fat Consumption	Troy, D. J.; Tiwari, B. K.; Joo, S. T.	2016	Other: Food Science of	Korean J Food Sci Anim Resour	Ireland	Not applicable (e.g. no	Review	Beef	General health	Positive	Unclear/Undeclar		Undeclared	Undeclared			Positive	Positive	Unclear/Undeclared	Unclear/Undeclar	Undeclared	Undeclared	Unclear/Undeclared	
Associations of plant and animal protein intake with 5-year changes in blood pressure: The Zutphen Elderly Study	Tielemans, S. M.; Kromhout, D.; Altorf-van der Kuil, W.; Geleijnse, J. M.	2014	Nutrition	Nutr Metab Cardiovasc Dis	Netherlands	Netherlands	Observational study	Animal protein	Cardiovascular disease	Neutral/mixed	Non-industry		Yes	Undeclared	Conflict of Interest Statement:		Neutral/mixed	Negative/neutral	Non-industry	No	No	Undeclared	Unclear/Undeclared	60
Investigating the risk-benefit balance of substituting red and processed meat with fish in a Danish diet	Thomsen, S. T.; Pires, S. M.; Devleesschauwer, B.; Poulsen, M.; Fagt, S.; Ygil, K. H.;	2018	Other: Food and Chemical	Food and Chemical Toxicology	Denmark	Denmark	Other: Modeling study	Red meat; Processed	Mortality; Nutritional outcomes; General	Negative	Non-industry		No	Non-industry			Negative	Negative/neutral	Non-industry	No	No	No	No	
Patterns of plant and animal protein intake are strongly associated with cardiovascular mortality: the Adventist Health Study-2	M. Tharrey, F. Mariotti, A. Mashchak, P. Barbillon, M. Delattre and G. E. Fraser	2018	Other: Epidemiology	Int J Epidemiol	United States	United States	Observational study	Animal protein	Cardiovascular disease; Mortality	Negative	Non-industry		No	Non-industry			Negative	Negative/neutral	Non-industry	No	No	No	No	100
Relative to processed red meat, alternative protein sources are associated with a lower risk of hypertension and diabetes in a	Thau, U.; Lajous, M.; Laouali, N.; Severi, G.; Boutron-Ruault, M. C.; MacDonald, C. J.	2021	Nutrition	Br J Nutr	France	France	Observational study	Processed meat	Cardiovascular disease; Diabetes	Negative	Non-industry		No	Non-industry			Negative	Negative/neutral	Non-industry	No	No	No	No	80
Are meat and heme iron intake associated with pancreatic cancer? Results from the NIH-AARP diet and health cohort	Taunk, P.; Hecht, E.; Stolzenberg-Solomon, R.	2016	Disease specific	Int J Cancer	United States	United States	Observational study	All meat	Cancer	Negative	Non-industry		No	Non-industry			Negative	Negative/neutral	Non-industry	No	No	No	No	100
Meat, Dietary Heme Iron, and Risk of Type 2 Diabetes Mellitus: The Singapore Chinese Health Study	Talaei, M.; Wang, Y. L.; Yuan, J. M.; Pan, A.; Koh, W. P.	2017	Other: Epidemiology	Am J Epidemiol	Singapore	Singapore	Observational study	All meat	Diabetes	Negative	Non-industry		No	Non-industry			Negative	Negative/neutral	Non-industry	No	No	No	No	100
A Joint Modeling Approach for Childhood Meat, Fish and Egg Consumption and the Risk of Advanced Islet Autoimmunity	SyrjÃ¤lÃ¤, E.; Nevalainen, J.; Peltonen, J.; Takkinen, H. M.; Hakola, L.; Ã…kerlund,	2019	Other: Science	Sci Rep	Finland	Finland	Observational study	All meat	Diabetes; Other: Advanced Islet	Negative	Non-industry		No	Non-industry			Negative	Negative/neutral	Non-industry	No	No	No	No	80
Red and processed meat and pancreatic cancer risk: a meta-analysis	Yudi Sun, Xinyi He, Yan Sun	2023	Nutrition	Front Nutr	China	Global	Meta analysis	Red meat; Processed	Cancer	Positive	Non-industry		No	Undeclared			Positive	Positive	Non-industry	No	No	Undeclared	Unclear/Undeclared	60
Red Meat Consumption and Risk for Cardio-Cerebrovascular Disease in Chinese Older Adults	Xiaojia Sun, Ruihong Sun, Liming Zhang	2023	Disease specific	Int Heart J	China	China	Observational study	Red meat	Cardiovascular disease	Neutral/mixed	Non-industry		No	Undeclared			Neutral/mixed	Negative/neutral	Non-industry	No	No	Undeclared	Unclear/Undeclared	40
Comparison of the effects of diets high in animal or plant protein on metabolic and cardiovascular markers in type 2 diabetes: A	Sucher, S.; Markova, M.; Hornemann, S.; Pivovarova, O.; Rudovich, N.; Thomann,	2017	Disease specific	Diabetes Obes Metab	Germany	Germany	Experimental study	Animal protein	Diabetes	Neutral/mixed	Unclear/Undeclar		No	Non-industry			Neutral/mixed	Negative/neutral	Unclear/Undeclared	Unclear/Undeclar	No	No	Unclear/Undeclared	80
Meat Consumption, Related Nutrients, Obesity and Risk of Prostate Cancer: a Case-Control Study in Uruguay	Stefani, E. D.; Boffetta, P.; Ronco, A. L.; Deneo-Pellegrini, H.;	2016	Disease specific	Asian Pac J Cancer Prev	Uruguay	Uruguay	Observational study	All meat	Cancer	Negative	Non-industry		Undeclared	Undeclared			Negative	Negative/neutral	Non-industry	No	Undeclared	Undeclared	Unclear/Undeclared	100
Meat consumption and stomach cancer risk: a meta-analysis	Song, P.; Lu, M.; Yin, Q.; Wu, L.; Zhang, D.; Fu, B.; Wang, B.; Zhao, Q.	2014	Disease specific	J Cancer Res Clin Oncol	China	Global	Meta analysis	Red meat	Cancer	Negative	Non-industry		No	Undeclared			Negative	Negative/neutral	Non-industry	No	No	Undeclared	Unclear/Undeclared	80
Association of Animal and Plant Protein Intake With All-Cause and Cause-Specific Mortality	Song, M.; Fung, T. T.; Hu, F. B.; Willett, W. C.; Longo, V. D.; Chan, A. T.; Giovannucci,	2016	Medicine broadly	JAMA Intern Med	United States	United States	Observational study	Animal protein	Mortality	Negative	Non-industry		No	Non-industry			Negative	Negative/neutral	Non-industry	No	No	No	No	100
Effects of processed red meat consumption on the risk of type 2 diabetes and cardiovascular diseases among Korean adults: the	Son, J.; Lee, Y.; Park, K.	2019	Nutrition	European Journal of Nutrition	South Korea	South Korea	Observational study	Processed meat	Cardiovascular disease; Diabetes	Neutral/mixed	Non-industry		No	Non-industry			Neutral/mixed	Negative/neutral	Non-industry	No	No	No	No	100
Red Meat Consumption and its Relationship With Cardiovascular Health: A Review of Pathophysiology and Literature	B. Singh, A. A. Khan, F. Anamika, R. Munjal, J. Munjal and R. Jain	2023	Other: Cardiology	Cardiol Rev	India	Not applicable (e.g. no	Review	Red meat	Cardiovascular disease	Negative	Non-industry		No	Undeclared			Negative	Negative/neutral	Non-industry	No	No	Undeclared	Unclear/Undeclared	
The impact of reduced red and processed meat consumption on cardiovascular risk factors; an intervention trial in healthy	Simpson, E. J.; Clark, M.; Razak, A. A.; Salter, A.	2019	Other: Food	Food Funct	UK	UK	Experimental study	Red meat; Processed	Cardiovascular disease	Neutral/mixed	Non-industry		No	Non-industry			Neutral/mixed	Negative/neutral	Non-industry	No	No	No	No	40
Dietary Intakes of Animal and Plant Proteins and Risk of Colorectal Cancer: The EPIC-Italy Cohort	Sieri, S.; Agnoli, C.; Pala, V.; Grioni, S.; Palli, D.; Bendinelli, B.; Macciotta, A.; Ricceri,	2022	Disease specific	Cancers (Basel)	Italy	Italy	Observational study	Animal protein	Cancer	Neutral/mixed	Non-industry		No	Non-industry			Neutral/mixed	Negative/neutral	Non-industry	No	No	No	No	60
Association of Meat Intake with Overweight and Obesity among School-aged Children and Adolescents	Shin, S. M.	2017	Disease specific	J Obes Metab Syndr	South Korea	South Korea	Observational study	All meat	Obesity	Positive	Non-industry		No	Undeclared			Positive	Positive	Non-industry	No	No	Undeclared	Unclear/Undeclared	40
Red meat consumption, cardiovascular diseases, and diabetes: a systematic review and meta-analysis	W. Shi, X. Huang, C. M. Schooling and J. V. Zhao	2023	Disease specific	Eur Heart J	China	Global	Systematic review	Red meat; Processed	Cardiovascular disease; Diabetes	Negative	Non-industry		No	Non-industry			Negative	Negative/neutral	Non-industry	No	No	No	No	60
Plant and animal protein intake and its association with depression, anxiety, and stress among Iranian women	Sheikhi, A.; Siassi, F.; Djazayery, A.; Guilani, B.; Azadbakht, L.	2023	Medicine broadly	BMC Public Health	Iran	Iran	Observational study	Animal protein	Other: Mental health	Negative	Non-industry		No	Non-industry			Negative	Negative/neutral	Non-industry	No	No	No	No	80
	Sheehy, S.; Palmer, J. R.; Rosenberg, L.	2020	Nutrition	J Nutr	United States	United States	Observational study	Red meat	Cardiovascular disease; Mortality	Negative	Non-industry		No	Non-industry			Negative	Negative/neutral	Non-industry	No	No	No	No	60
Animal Protein Intake Is Associated with General Adiposity in Adolescents: The Teen Food and Development Study	Segovia-Siapco, G.; Khayef, G.; Pribis, P.; Oda, K.; Haddad, E.; SabatÃ©, J.	2020	Nutrition	Nutrients	United States	United States	Observational study	Animal protein	Obesity	Negative	Non-industry		No	No funding received			Negative	Negative/neutral	Non-industry	No	No	No	No	80
Association of Red Meat Intake with the Risk of Cardiovascular Mortality in General Japanese Stratified by Kidney Function:	Segawa, H.; Kondo, K.; Kadota, A.; Yamauchi, H.; Ohno, S.; Tanaka-Mizuno, S.;	2020	Nutrition	Nutrients	Japan	Japan	Observational study	Red meat	Cardiovascular disease; Mortality; Other:	Positive	Non-industry		No	Non-industry			Positive	Positive	Non-industry	No	No	No	No	80
Protein intake and type 2 diabetes mellitus: an umbrella review of systematic reviews for the evidence-based guideline for protein	Schulze, M. B.; Haardt, J.; Amini, A. M.; Kalotai, N.; Lehmann, A.; Schmidt, A.;	2023	Nutrition	European Journal of Nutrition	Germany	Global	Systematic review	Animal protein	Diabetes	Neutral/mixed	Non-industry		Yes	Non-industry			Neutral/mixed	Negative/neutral	Non-industry	No	No	No	No	100
Equivalent reductions in body weight during the Beef WISE Study: beef's role in weight improvement, satisfaction and energy	Sayer, R. D.; Speaker, K. J.; Pan, Z.; Peters, J. C.; Wyatt, H. R.; Hill, J. O.	2017	Disease specific	Obes Sci Pract	United States	United States	Experimental study	Beef	Obesity	Neutral/mixed	Non-industry		Yes	Industry	Beef Checkoff	Not involved in either	Neutral/mixed	Negative/neutral	Non-industry	No	Yes	Yes	Yes	60
	Saneei, P.; Willett, W.; Esmaillzadeh, A.	2015	Medicine broadly	J Res Med Sci	Iran	Global	Systematic review	Red meat; Processed	Cancer	Neutral/mixed	Non-industry		No	Non-industry			Neutral/mixed	Negative/neutral	Non-industry	No	No	No	No	100
A case-control study on red meat consumption and risk of stroke among a group of Iranian adults	Saneei, P.; Saadatnia, M.; Shakeri, F.; Beykverdi, M.; Keshteli, A. H.; Esmaillzadeh,	2015	Nutrition	Public Health Nutrition	Iran	Iran	Observational study	Red meat	Cardiovascular disease	Negative	Non-industry		No	Non-industry			Negative	Negative/neutral	Non-industry	No	No	No	No	100
Red meat and processed meat consumption and risk factors for type 2 diabetes: a systematic review and meta-analysis of randomized controlled trials	Sanders, L. M.; Wilcox, M. L.; Maki, K. C.	2023	Nutrition	Eur J Clin Nutr	United States	Global	Systematic review	Red meat	Diabetes	Positive	Industry	Midwest Biomedical	Yes	Industry	Beef Checkoff	Involved in design	Positive	Positive	Industry	Yes	Yes	Yes	Yes	60
Red and processed meat intake and risk of colorectal cancer: A population-based case-control study	W. Saliba, H. S. Rennert, N. Gronich, S. B. Gruber and G. Rennert	2019	Disease specific	Eur J Cancer Prev	Israel	Israel	Observational study	All meat	Cancer	Neutral/mixed	Non-industry		No	Non-industry			Neutral/mixed	Negative/neutral	Non-industry	No	No	No	No	100
Association between meat intake and mortality due to all-cause and major causes of death in a Japanese population	Saito, E.; Tang, X.; Abe, S. K.; Sawada, N.; Ishihara, J.; Takachi, R.; Iso, H.; Shimazu,	2020	Medicine broadly	PLoS One	Japan	Japan	Observational study	All meat	Mortality	Neutral/mixed	Non-industry		No	Non-industry			Neutral/mixed	Negative/neutral	Non-industry	No	No	No	No	100
Unscrambling the relations of egg and meat consumption with type 2 diabetes risk	J. SabatÃ©, N. M. Burkholder-Cooley, G. Segovia-Siapco, K. Oda, B. Wells, M. J.	2018	Nutrition	American Journal of Clinical Nutrition	United States	United States	Observational study	All meat; Animal	Diabetes	Negative	Non-industry		No	Industry	American Egg Board	Not involved in either	Negative	Negative/neutral	Non-industry	No	No	No	No	100
Processed meat consumption and associated factors in Chile: A cross-sectional study nested in the MAUCO cohort	Ruedlinger, J.; Cid-OssandÃ³n, V.; Huidobro, A.; Van De Wyngard, V.; Vargas, C.;	2022	Medicine broadly	Frontiers in Public Health	Chile	Chile	Observational study	Processed meat	Obesity; Cardiovascular disease; Cancer	Negative	Non-industry		No	Non-industry			Negative	Negative/neutral	Non-industry	No	No	No	No	80
Intake of dietary fibre, red and processed meat and risk of late-onset Chronic Inflammatory Diseases: A prospective Danish study	Rubin, K. H.; Rasmussen, N. F.; Petersen, I.; Kopp, T. I.; Stenager, E.; Magyari, M.;	2020	Medicine broadly	Int J Med Sci	Denmark	Denmark	Observational study	Red meat; Processed	Cancer; Other: Chronic Inflammatory	Positive	Non-industry		Undeclared	Non-industry			Positive	Positive	Non-industry	No	Undeclared	No	Unclear/Undeclared	80
Estimates of the current and future burden of cancer attributable to red and processed meat consumption in Canada	Ruan, Y.; Poirier, A. E.; Hebert, L. A.; Grevers, X.; Walter, S. D.; Villeneuve, P. J.;	2019	Medicine broadly	Prev Med	Canada	Canada	Other: Modeling study	Red meat; Processed	Cancer	Negative	Non-industry		No	Non-industry			Negative	Negative/neutral	Non-industry	No	No	No	No	
Effects of a DASH-like diet containing lean beef on vascular health	Roussell, M. A.; Hill, A. M.; Gaugler, T. L.; West, S. G.; Ulbrecht, J. S.; Vanden	2014	Disease specific	J Hum Hypertens	United States	United States	Experimental study	Beef	Cardiovascular disease	Positive	Non-industry		Yes	Industry	Beef Checkoff	Undeclared	Positive	Positive	Non-industry	No	Yes	Yes	Yes	60
Is there a relationship between red or processed meat intake and obesity? A systematic review and meta-analysis of observational	M. H. Rouhani, A. Salehi-Abargouei, P. J. Surkan and L. Azadbakht	2014	Disease specific	Obesity Reviews	Iran	Global	Systematic review	Red meat; Processed	Obesity	Negative	Non-industry		No	Undeclared			Negative	Negative/neutral	Non-industry	No	No	Undeclared	Unclear/Undeclared	80
Processed Meat and Colorectal Cancer: A Pooled Analysis of Three Italian Case-Control Studies	Rosato, V.; Tavani, A.; Negri, E.; Serraino, D.; Montella, M.; Decarli, A.; La Vecchia,	2017	Nutrition	Nutr Cancer	Italy	Italy	Meta analysis	Processed meat	Cancer	Neutral/mixed	Non-industry		Undeclared	Non-industry			Neutral/mixed	Negative/neutral	Non-industry	No	Undeclared	No	Unclear/Undeclared	
Processed Meat and Risk of Renal Cell and Bladder Cancers	Rosato, V.; Negri, E.; Serraino, D.; Montella, M.; Libra, M.; Lagiou, P.; Facchini, G.;	2018	Nutrition	Nutr Cancer	Italy	Italy	Observational study	Processed meat	Cancer	Neutral/mixed	Non-industry		Undeclared	Non-industry			Neutral/mixed	Negative/neutral	Non-industry	No	Undeclared	No	Unclear/Undeclared	80
Processed meat and selected hormone-related cancers	V. Rosato, E. Negri, F. Parazzini, D. Serraino, M. Montella, M. Ferraroni, et al.	2018	Nutrition	Nutrition	Italy	Italy	Review	Processed meat	Cancer	Neutral/mixed	Non-industry		Yes	Non-industry			Neutral/mixed	Negative/neutral	Non-industry	No	No	No	No	
Processed meat and risk of selected digestive tract and laryngeal cancers	Rosato, V.; Kawakita, D.; Negri, E.; Serraino, D.; Garavello, W.; Montella, M.;	2019	Nutrition	Eur J Clin Nutr	Italy	Italy	Observational study	Processed meat	Cancer	Neutral/mixed	Non-industry		No	Non-industry			Neutral/mixed	Negative/neutral	Non-industry	No	No	No	No	80
Meat consumption, animal products, and the risk of bladder cancer: a case-control study in Uruguayan men	Ronco, A. L.; Mendilaharsu, M.; Boffetta, P.; Deneo-Pellegrini, H.; De Stefani, E.	2014	Disease specific	Asian Pac J Cancer Prev	Uruguay	Uruguay	Observational study	All meat	Cancer	Negative	Non-industry		Undeclared	Undeclared			Negative	Negative/neutral	Non-industry	No	Undeclared	Undeclared	Unclear/Undeclared	100
Meat and fish consumption and the risk of renal cell carcinoma in the European prospective investigation into cancer and nutrition	Rohrmann, S.; Linseisen, J.; Overvad, K.; WÃ¼rtz, A. M. L.; Roswall, N.; Tjonneland,	2015	Disease specific	Int J Cancer	Switzerland	Europe	Observational study	All meat	Cancer	Negative	Non-industry		Yes	Undeclared			Negative	Negative/neutral	Non-industry	No	No	Undeclared	Unclear/Undeclared	80
Processed meat: the real villain?	Rohrmann, S.; Linseisen, J.	2016	Nutrition	Proc Nutr Soc	Switzerland	Not applicable (e.g. no	Review	Processed meat	General health	Neutral/mixed	Non-industry		No	Non-industry			Neutral/mixed	Negative/neutral	Non-industry	No	No	No	No	
SWAP-MEAT Athlete (study with appetizing plant-food, meat eating alternatives trial) - investigating the impact of three different	Roberts, A. K.; Busque, V.; Robinson, J. L.; Landry, M. J.; Gardner, C. D.	2022	Nutrition	Nutrition Journal	United States	United States	Experimental study	All meat; Other: Meat	Muscle mass; Other: Athletic performance	Neutral/mixed	Non-industry		Yes	Non-industry			Neutral/mixed	Negative/neutral	Non-industry	No	No	No	No	60
The current and future costs of colorectal cancer attributable to red and processed meat consumption in Brazil	Rezende, L. F. M.; MalhÃ£o, T. A.; da Silva Barbosa, R.; Schilithz, A. O. C.; da Silva,	2021	Medicine broadly	BMC Health Serv Res	Brazil	Brazil	Modeling study	Red meat; Processed	Cancer	Negative	Non-industry		No	Non-industry			Negative	Negative/neutral	Non-industry	No	No	No	No	
Influence of Alcohol and Red Meat Consumption on Life Expectancy: Results of 164 Countries from 1992 to 2013	C. L. Ranabhat, M. B. Park and C. B. Kim	2020	Nutrition	Nutrients	South Korea	Global	Observational study	Red meat	Mortality	Negative	Non-industry		No	Non-industry			Negative	Negative/neutral	Non-industry	No	No	No	No	80
Associations of Meat and Fish Consumption With Conventional and Radiomics Cardiovascular Magnetic Resonance Phenotypes in	Z. Raisi-Estabragh, C. McCracken, P. Gkontra, A. Jaggi, M. Ardissino, J. Cooper, et al.	2021	Disease specific	Front Cardiovasc Med	UK	UK	Observational study	Red meat; Processed	Cardiovascular disease	Negative	Non-industry		No	Undeclared			Negative	Negative/neutral	Non-industry	No	No	Undeclared	Unclear/Undeclared	80
Red and Processed Meat Intake in Relation to Non-Alcoholic Fatty Liver Disease Risk: Results from a Case-Control Study	F. Rahimi-Sakak, M. Maroofi, H. Emamat and A. Hekmatdoost	2022	Nutrition	Clin Nutr Res	Iran	Iran	Observational study	Red meat; Processed	Other: Liver disease	Negative	Non-industry		No	Non-industry			Negative	Negative/neutral	Non-industry	No	No	No	No	
Red and processed meats and health risks: how strong is the evidence?	F. Qian, M. C. Riddle, J. Wylie-Rosett and F. B. Hu	2020	Disease specific	Diabetes Care	United States	Not applicable (e.g. no	Review	Red meat; Processed	General health	Negative	Non-industry		Yes	Non-industry			Negative	Negative/neutral	Non-industry	No	No	No	No	
Lack of Association between Red Meat Consumption and a Positive Fecal Immunochemical Colorectal Cancer Screening Test in	P. Pramual, P. Sarakarn, S. Kamsa-ard, C. Jirapornkul, N. Maneenin, P.	2018	Disease specific	Asian Pac J Cancer Prev	Thailand	Thailand	Experimental study	Red meat	Cancer	Positive	Non-industry		No	Undeclared			Positive	Positive	Non-industry	No	No	Undeclared	Unclear/Undeclared	20
The Associations between Meat Group Consumption and Acute Myocardial Infarction Risks in an Iranian Population: a Case-	Z. Poursafar, F. Joukar, F. Hasavari and Z. Atrkar Roushan	2019	Nutrition	Clin Nutr Res	Iran	Iran	Observational study	Poultry; Red meat;	Cardiovascular disease	Negative	Non-industry		No	Undeclared			Negative	Negative/neutral	Non-industry	No	No	Undeclared	Unclear/Undeclared	80
The Association of Consumption of Animal Proteins and the Risk of Esophageal Cancer	S. J. Pourmaghi, F. Barazandeh Noveyri, H. Mohammad Doust, A. Ahmadi, A.	2018	Other: Nutrition and Cancer	Nutr Cancer	Iran	Iran	Observational study	All meat	Cancer	Negative	Non-industry		No	Non-industry			Negative	Negative/neutral	Non-industry	No	No	No	No	80
Influence of Weight Reduction and Enhanced Protein Intake on Biomarkers of Inflammation in Older Adults with Obesity	K. N. Porter Starr, M. Orenduff, S. R. McDonald, H. Mulder, R. Sloane, C. F. Pieper	2019	Nutrition	J Nutr Gerontol Geriatr	United States	United States	Experimental study	Beef; Animal protein	Obesity; Other: Inflammation Biomarkers	Positive	Non-industry		Undeclared	Industry	Beef Checkoff	Not involved in either	Positive	Positive	Non-industry	No	Undeclared	Yes	Yes	20
Impact on cardiometabolic risk of a weight loss intervention with higher protein from lean red meat: Combined results of 2	K. N. Porter Starr, M. A. Connelly, M. C. Orenduff, S. R. McDonald, R. Sloane, K. M.	2019	Other: Clinical Lipidology	J Clin Lipidol	United States	United States	Experimental study	Red meat	Obesity; Cardiovascular disease	Positive	Unclear/Undeclar		Yes	Industry	Beef Checkoff, Pork Checkoff, Nort	Not involved in either	Positive	Positive	Unclear/Undeclared	Unclear/Undeclar	No	Yes	Yes	80
Effects of diets high in animal or plant protein on oxidative stress in individuals with type 2 diabetes: A randomized clinical trial	O. Pivovarova-Ramich, M. Markova, D. Weber, S. Sucher, S. Hornemann, N.	2020	Other: Biology	Redox Biol	Germany	Germany	Experimental study	Animal protein	Diabetes	Neutral/mixed	Non-industry		No	Non-industry			Neutral/mixed	Negative/neutral	Non-industry	No	No	No	No	40
Meat consumption and colorectal cancer: an evaluation based on a systematic review of epidemiologic evidence among the	Pham, N. M.; Mizoue, T.; Tanaka, K.; Tsuji, I.; Tamakoshi, A.; Matsuo, K.; et al.	2014	Disease specific	Jpn J Clin Oncol	Japan	Japan	Systematic review	All meat	Cancer	Negative	Non-industry		No	Non-industry			Negative	Negative/neutral	Non-industry	No	No	No	No	60
A prospective analysis of intake of red and processed meat in relation to pancreatic cancer among African American Women	J. L. Petrick, N. Castro-Webb, H. Gerlovin, T. N. Bethea, S. Li, E. A. Ruiz-Narvaez, et	2020	Disease specific	Cancer Epidemiol Biomarkers Prev	United States	United States	Observational study	Red meat; Processed	Cancer	Negative	Non-industry		No	Non-industry			Negative	Negative/neutral	Non-industry	No	No	No	No	
Western and Carnivorous Dietary Patterns are Associated with Greater Likelihood of IBD Development in a Large Prospective	V. Peters, L. Bolte, E. Schuttert, S. Andreu-SÃ¡nchez, G. Dijkstra, R. Weersma and M.	2022	Disease specific	J Crohns Colitis	Netherlands	Netherlands	Observational study	All meat	Other: Inflammatory Bowel Disease	Negative	Non-industry		Yes	Non-industry			Negative	Negative/neutral	Non-industry	No	No	No	No	100
Vegetarians, fish, poultry, and meat-eaters: who has higher risk of cardiovascular disease incidence and mortality? A prospective	F. Petermann-Rocha, S. Parra-Soto, S. Gray, J. Anderson, P. Welsh, J. Gill, et al.	2021	Disease specific	Eur Heart J	UK	UK	Observational study	All meat	Cardiovascular disease	Negative	Non-industry		Yes	Non-industry			Negative	Negative/neutral	Non-industry	No	No	No	No	80
A calorie-restricted DASH diet reduces body fat and maintains muscle strength in obese older adults	C. A. Perry, G. P. Van Guilder, A. Kauffman and M. Hossain	2019	Nutrition	Nutrients	United States	United States	Experimental study	Red meat	Obesity; Muscle mass	Positive	Non-industry		No	Industry	South Dakota Beef Industry Council	Undeclared	Positive	Positive	Non-industry	No	No	Yes	Yes	60
Intake of meat and fish and risk of head-neck cancer subtypes in the Netherlands Cohort Study	A. Perloy, D. H. E. Maasland, P. A. van den Brandt, B. Kremer and L. J. Schouten	2017	Disease specific	Cancer Causes Control	Netherlands	Netherlands	Observational study	All meat	Cancer	Negative	Non-industry		No	Non-industry			Negative	Negative/neutral	Non-industry	No	No	No	No	100
Association of meat consumption with NAFLD risk and liver-related biochemical indexes in older Chinese: a cross-sectional study	H. Peng, X. Xie, X. Pan, J. Zheng, Y. Zeng, X. Cai, et al.	2021	Other: Gastroenterology	BMC Gastroenterol	China	China	Observational study	All meat	Other: Liver disease	Negative	Non-industry		No	Non-industry			Negative	Negative/neutral	Non-industry	No	No	No	No	100
Reï¬‚placing dietary animal-source proteins with plant-source proteins changes dietary intake and status of vitamins and minerals	T. Pellinen, E. PÃ¤ivÃ¤rinta, J. Isotalo, M. Lehtovirta, S. T. Itkonen, L. Korkalo, et al.	2022	Nutrition	European Journal of Nutrition	Finland	Finland	Experimental study	Animal protein	Nutritional outcomes; Other: Vitamin B,	Positive	Non-industry		Yes	Industry	Raisio Plcâ€™s Research Foundation,	Undeclared	Positive	Positive	Non-industry	No	No	Yes	Yes	60

Association of meat, vegetarian, pescatarian and fish-poultry diets with risk of 19 cancer sites and all cancer: findings from the UK	S. Parra-Soto, D. Ahumada, F. Petermann-Rocha, J. Boonpoor, J. L. Gallegos, J.	2022	Medicine broadly	BMC Med	UK	UK	Observational study	All meat	Cancer	Negative	Non-industry		No	Non-industry			Negative	Negative/neutral	Non-industry	No	No	No	No	80
Unprocessed Meat Consumption and Incident Cardiovascular Diseases in Korean Adults: The Korean Genome and Epidemiology	K. Park, J. Son, J. Jang, R. Kang, H. K. Chung, K. W. Lee, et al.	2017	Nutrition	Nutrients	South Korea	South Korea	Observational study	All meat	Cardiovascular disease	Positive	Non-industry		No	Non-industry			Positive	Positive	Non-industry	No	No	No	No	100
Grilled, Barbecued, and Smoked Meat Intake and Survival Following Breast Cancer	H. Parada, S. E. Steck, P. T. Bradshaw, L. S. Engel, K. Conway, S. L. Teitelbaum, et al.	2017	Disease specific	J Natl Cancer Inst	United States	United States	Observational study	All meat	Mortality; Cancer	Negative	Non-industry		Undeclared	Non-industry			Negative	Negative/neutral	Non-industry	No	Undeclared	No	Unclear/Undeclared	80
Association of poultry consumption with cardiovascular diseases and all-cause mortality: a systematic review and dose response	R. E. Papp, V. Hasenegger, C. Ekmekcioglu and L. Schwingshackl	2021	Nutrition	Crit Rev Food Sci Nutr	Austria	Global	Systematic review	Poultry	Cardiovascular disease; Mortality	Neutral/mixed	Non-industry		No	No funding received			Neutral/mixed	Negative/neutral	Non-industry	No	No	No	No	80
	K. Papier, A. Knuppel, N. Syam, S. A. Jebb and T. J. Key	2023	Nutrition	Crit Rev Food Sci Nutr	UK	Global	Systematic review	Poultry; Red meat;	Ischemic Heart Disease	Negative	Non-industry		No	Non-industry			Negative	Negative/neutral	Non-industry	No	No	No	No	100
Meat consumption and risk of 25 common conditions: outcome-wide analyses in 475,000 men and women in the UK Biobank	K. Papier, G. K. Fensom, A. Knuppel, P. N. Appleby, T. Y. N. Tong, J. A. Schmidt, et al.	2021	Medicine broadly	BMC Med	UK	UK	Observational study	All meat	General health	Neutral/mixed	Non-industry		No	Non-industry			Neutral/mixed	Negative/neutral	Non-industry	No	No	No	No	100
Association of Red Meat Consumption, Metabolic Markers, and Risk of Cardiovascular Diseases	L. Pan, L. Chen, J. Lv, Y. Pang, Y. Guo, P. Pei, et al.	2022	Nutrition	Front Nutr	China	China	Observational study	Red meat	Cardiovascular disease; Other: Metabolic	Negative	Non-industry		No	Non-industry			Negative	Negative/neutral	Non-industry	No	No	No	No	100
Protein Intake by Source and Breast Cancer Incidence and Mortality: The Women'sâ€‚Health Initiative	K. Pan, J. C. Larson, R. L. Prentice, J. E. Mortimer, M. L. Neuhouser, J. E. Manson, et	2020	Disease specific	JNCI Cancer Spectr	United States	United States	Observational study	Animal protein	Cancer	Negative	Non-industry		Yes	Non-industry			Negative	Negative/neutral	Non-industry	No	No	No	No	60
A Lean Pork-Containing Breakfast Reduces Hunger and Glycemic Response Compared to a Refined Carbohydrate-Containing	O. M. Palacios, I. Edirisinghe, M. L. Wilcox, B. Burton-Freeman, D. Xiao and K. C.	2018	Nutrition	J Am Coll Nutr	United States	United States	Experimental study	Pork	Diabetes	Positive	Non-industry		Yes	Industry	National Pork Board.	Undeclared	Positive	Positive	Non-industry	No	Yes	Yes	Yes	40
High Dietary Intake of Vegetable Protein Is Associated With Lower Prevalence of Renal Function Impairment: Results of the Dutch	M. M. Oosterwijk, S. S. Soedamah-Muthu, J. M. Geleijnse, S. J. L. Bakker, G. Navis, S.	2019	Disease specific	Kidney Int Rep	Netherlands	Netherlands	Observational study	Animal protein	Other: Kidney disease	Negative	Non-industry		Yes	Non-industry			Negative	Negative/neutral	Non-industry	No	No	No	No	100
A Mediterranean-style eating pattern with lean, unprocessed red meat has cardiometabolic benefits for adults who are overweight		2018	Nutrition	American Journal of Clinical Nutrition	United States	United States	Experimental study	Red meat	Obesity; Cardiovascular disease	Positive	Non-industry		Yes	Industry	Beef Checkoff, Pork Checkoff	Not involved in either	Positive	Positive	Non-industry	No	Yes	Yes	Yes	60
Effects of Total Red Meat Intake on Glycemic Control and Inflammatory Biomarkers: A Meta-Analysis of Randomized Controlled	L. E. O'Connor, J. E. Kim, C. M. Clark, W. B. Zhu and W. W. Campbell	2021	Nutrition	Adv Nutr	United States	Global	Meta analysis	Red meat	Other: Glycemic Control and Inflammatory	Positive	Non-industry		Yes	Industry	Pork Checkoff	Not involved in either	Positive	Positive	Non-industry	No	Yes	Yes	Yes	80
Total red meat intake of â‰¥0.5 servings/d does not negatively influence cardiovascular disease risk factors: A systemically	L. E. O'Connor, J. E. Kim and W. W. Campbell	2017	Nutrition	American Journal of Clinical Nutrition	United States	Global	Meta analysis	Red meat	Cardiovascular disease	Positive	Non-industry		Yes	Non-industry			Positive	Positive	Non-industry	No	Yes	No	Yes	100
Adopting a Mediterranean-Style Eating Pattern with Different Amounts of Lean Unprocessed Red Meat Does Not Influence Short-	L. E. O'Connor, S. L. Biberstine, D. Paddon-Jones, A. J. Schwichtenberg and W. W.	2018	Nutrition	J Nutr	United States	United States	Experimental study	Red meat	General health; Other: Mental health	Neutral/mixed	Non-industry		Yes	Industry	Beef Checkoff, Pork Checkoff	Not involved in either	Neutral/mixed	Negative/neutral	Non-industry	No	Yes	Yes	Yes	100
Red and Processed Meat Consumption and Risk of Depression: A Systematic Review and Meta-Analysis	D. Nucci, C. Fatigoni, A. Amerio, A. Odone and V. Gianfredi	2020	Other: Environmental	Int J Environ Res Public Health	Italy	Global	Systematic review	Red meat; Processed	Other: Mental Health	Negative	Non-industry		No	No funding received			Negative	Negative/neutral	Non-industry	No	No	No	No	100
Association Between Red and Processed Meat Consumption and Risk of Prostate Cancer: A Systematic Review and Meta-Analysis	S. Nouri-Majd, A. Salari-Moghaddam, A. Aminianfar, B. Larijani and A. Esmaillzadeh	2022	Nutrition	Front Nutr	Iran	Global	Systematic review	Red meat; Processed	Cancer	Negative	Non-industry		No	Undeclared			Negative	Negative/neutral	Non-industry	No	No	Undeclared	Unclear/Undeclared	100
Impact of Reducing Intake of Red and Processed Meat on Colorectal Cancer Incidence in Germany 2020 to 2050-A Simulation	T. Niedermaier, T. Gredner, M. Hoffmeister, U. Mons and H. Brenner	2023	Nutrition	Nutrients	Germany	Germany	Other: Modeling study	Red meat; Processed	Cancer	Negative	Non-industry		No	Non-industry			Negative	Negative/neutral	Non-industry	No	No	No	No	
Red meat consumption is associated with prediabetes and diabetes in rural Vietnam: A cross-sectional study	C. Q. Nguyen, T. T. P. Pham, A. Fukunaga, D. V. Hoang, T. V. Phan, D. C. Phan, et al.	2023	Nutrition	Public Health Nutrition	Vietnam	Vietnam	Observational study	Red meat	Diabetes	Neutral/mixed	Non-industry		No	Industry	Pfizer Health Research Foundation	Not involved in either	Neutral/mixed	Negative/neutral	Non-industry	No	No	No	No	100
Nutrient Intake and Status in Children and Adolescents Consuming Plant-Based Diets Compared to Meat-Eaters: A Systematic	N. Neufingerl and A. Eilander	2023	Nutrition	Nutrients	Netherlands	Global	Systematic review	All meat	Nutritional outcomes	Neutral/mixed	Industry	Unilever	Yes	No funding received			Neutral/mixed	Negative/neutral	Industry	No	No	No	No	60
Nutrient Intake and Status in Adults Consuming Plant-Based Diets Compared to Meat-Eaters: A Systematic Review	N. Neufingerl and A. Eilander	2021	Nutrition	Nutrients	Netherlands	Global	Systematic review	All meat	Nutritional outcomes	Neutral/mixed	Industry	Unilever	Yes	No funding received			Neutral/mixed	Negative/neutral	Industry	No	No	No	No	40
Protein supplementation with low fat meat after resistance training: effects on body composition and strength	M. Negro, M. Vandoni, S. Ottobrini, E. Codrons, L. Correale, D. Buonocore and F.	2014	Nutrition	Nutrients	Italy	Italy	Experimental study	Beef	Muscle mass	Positive	Non-industry		No	Undeclared			Positive	Positive	Non-industry	No	No	Undeclared	Unclear/Undeclared	20
Dietary intake of total, animal, and plant proteins and risk of all cause, cardiovascular, and cancer mortality: systematic review and	S. Naghshi, O. Sadeghi, W. C. Willett and A. Esmaillzadeh	2020	Medicine broadly	BMJ	Iran	Global	Systematic review	Animal protein	Cardiovascular disease; Mortality; Cancer	Neutral/mixed	Non-industry		No	Non-industry			Neutral/mixed	Negative/neutral	Non-industry	No	No	No	No	100
A comparison of regular consumption of fresh lean pork, beef and chicken on body composition: A randomized cross-over trial	K. J. Murphy, B. Parker, K. A. Dyer, C. R. Davis, A. M. Coates, J. D. Buckley and P. R.	2014	Nutrition	Nutrients	Australia	Australia	Experimental study	Beef; Pork; Poultry	Obesity	Positive	Non-industry		No	Industry	Australian Pork Ltd., Pork Co-	Undeclared	Positive	Positive	Non-industry	No	No	Yes	Yes	20
Meat consumption and breast cancer: a case-control study in women	N. Mourouti, M. D. Kontogianni, C. Papavagelis, P. Plytzanopoulou, T. Vassilakou, T.	2015	Other: Meat Science	Meat Science	Greece	Greece	Observational study	All meat	Cancer	Neutral/mixed	Non-industry		No	No funding received			Neutral/mixed	Negative/neutral	Non-industry	No	No	No	No	100
Meat intake, meat cooking methods, and meat-derived mutagen exposure and risk of sessile serrated lesions	D. Mosley, T. Su, H. J. Murff, W. E. Smalley, R. M. Ness, W. Zheng and M. J.	2020	Nutrition	American Journal of Clinical Nutrition	United States	United States	Observational study	All meat	Cancer	Negative	Non-industry		No	Non-industry			Negative	Negative/neutral	Non-industry	No	No	No	No	100
Beneficial Impact of Pork Dry-Cured Ham Consumption on Blood Pressure and Cardiometabolic Markers in Individuals with	S. Montoro-GarcÃa, Ã Velasco-Soria, L. Mora, C. Carazo-DÃaz, D. Prieto-Merino,	2022	Nutrition	Nutrients	Spain	Spain	Experimental study	Pork	Cardiovascular disease	Positive	Industry	ElPozo AlimentaciÃ³n	No	Non-industry			Positive	Positive	Industry	Yes	No	No	Yes	80
Ultraprocessed beverages and processed meats increase the incidence of hypertension in Mexican women	A. Monge, D. Silva Canella, N. LÃ³pez-Olmedo, M. Lajous, A. CortÃ©s-Valencia and	2021	Nutrition	Br J Nutr	Mexico	Mexico	Observational study	Processed meat	Cardiovascular disease	Negative	Non-industry		No	Non-industry			Negative	Negative/neutral	Non-industry	No	No	No	No	100
Meat consumption, stratified by dietary quality, and risk of heart disease	S. P. Moller, H. Mejborn, A. I. Christensen, A. Biltoft-Jensen and L. C. Thygesen	2021	Nutrition	Br J Nutr	Denmark	Denmark	Observational study	All meat	Cardiovascular disease	Neutral/mixed	Non-industry		No	Non-industry			Neutral/mixed	Negative/neutral	Non-industry	No	No	No	No	80
The association of dietary animal and plant protein with putative risk markers of colorectal cancer in overweight pre-diabetic	G. MÃ¸ller, J. R. Andersen, E. Jalo, C. Ritz, J. Brand-Miller, T. M. Larsen, et al.	2020	Nutrition	European Journal of Nutrition	Denmark	Global	Observational study	Animal protein	Obesity; Cancer; Diabetes	Neutral/mixed	Non-industry		Yes	Industry	Danish Technological Institute, The	Undeclared	Neutral/mixed	Negative/neutral	Non-industry	No	No	Yes	Yes	60
Red Meat Consumption (Heme Iron Intake) and Risk for Diabetes and Comorbidities?	R. Misra, P. Balagopal, S. Raj and T. G. Patel	2018	Disease specific	Curr Diab Rep	United States	Not applicable (e.g. no	Review	Red meat	Diabetes	Negative	Non-industry		No	Undeclared			Negative	Negative/neutral	Non-industry	No	No	Undeclared	Unclear/Undeclared	
A Prospective Study of Dietary Meat Intake and Risk of Incident Chronic Kidney Disease	P. Mirmiran, E. Yuzbashian, M. Aghayan, M. Mahdavi, G. Asghari and F. Azizi	2020	Nutrition	J Ren Nutr	Iran	Iran	Observational study	Red meat; Processed	Other: Kidney disease	Negative	Non-industry		No	Non-industry			Negative	Negative/neutral	Non-industry	No	No	No	No	60
Therapeutic lifestyle change diet enriched in legumes reduces oxidative stress in overweight type 2 diabetic patients: a crossover	P. Mirmiran, S. Hosseinpour-Niazi and F. Azizi	2018	Nutrition	Eur J Clin Nutr	Iran	Iran	Experimental study	Red meat	Obesity; Diabetes; Other: Oxidative stress	Negative	Non-industry		No	Undeclared			Negative	Negative/neutral	Non-industry	No	No	Undeclared	Unclear/Undeclared	100
Meat and colorectal cancer in Middle Eastern and North African countries: update of literature review	M. Mint Sidi Deoula, K. El Kinany, Z. Hatime, H. A. Boudouaya and K. El Rhazi	2020	Medicine broadly	Public Health Rev	Morocco	Middle East and North	Review	All meat	Cancer	Positive	Non-industry		No	Non-industry			Positive	Positive	Non-industry	No	No	No	No	
Associations of red and processed meat with survival among patients with cancers of the upper aerodigestive tract and lung	F. L. Miles, S. C. Chang, H. Morgenstern, D. Tashkin, J. Y. Rao, W. Cozen, et al.	2016	Nutrition	Nutr Res	United States	United States	Observational study	Red meat; Processed	Mortality; Cancer	Negative	Non-industry		No	Non-industry			Negative	Negative/neutral	Non-industry	No	No	No	No	60
Animal Protein Intake Is Inversely Associated With Mortality in Older Adults: The InCHIANTI Study	T. MeroÃ±o, R. Zamora-Ros, N. Hidalgo-Liberona, M. Rabassa, S. Bandinelli, L.	2022	Medicine broadly	J Gerontol A Biol Sci Med Sci	Spain	Italy	Observational study	Animal protein	Mortality	Positive	Non-industry		No	Non-industry			Positive	Positive	Non-industry	No	No	No	No	80
Associations between Dietary Animal and Plant Protein Intake and Cardiometabolic Risk Factors-A Cross-Sectional Study in China	S. Meng, Z. Cui, M. Li, T. Li, F. Wu, T. Kang and H. Meng	2021	Nutrition	Nutrients	China	China	Observational study	Animal protein	Cardiovascular disease	Neutral/mixed	Non-industry		No	Non-industry			Neutral/mixed	Negative/neutral	Non-industry	No	No	No	No	100
The nutritional profile of plant-based meat analogues available for sale in Australia	H. Melville, M. Shahid, A. Gaines, B. L. McKenzie, R. Alessandrini, K. Trieu, et al.	2023	Nutrition	Nutr Diet	Australia	Australia	Observational study	All meat; Other: Meat	Nutritional outcomes	Neutral/mixed	Non-industry		No	Industry	Marlow Foods LTD (alternative meat	Not involved in either	Neutral/mixed	Negative/neutral	Non-industry	No	No	No	No	
Dietary Intake of Red Meat, Processed Meat, and Poultry and Risk of Colorectal Cancer and All-Cause Mortality in the Context of	H. Mejborn, S. P. MÃ¸ller, LH. Mejborn, S. P. MÃ¸ller, L. C. Thygesen and A. Biltoft-	2020	Nutrition	Nutrients	Denmark	Denmark	Observational study	Poultry; Red meat;	Mortality; Cancer	Positive	Non-industry		No	Non-industry			Positive	Positive	Non-industry	No	No	No	No	100
A Prospective Analysis of Red and Processed Meat Consumption and Risk of Colorectal Cancer in Women	S. S. Mehta, W. D. Arroyave, R. M. Lunn, Y. M. M. Park, W. A. Boyd and D. P.	2019	Disease specific	Cancer Epidemiol Biomarkers Prev	United States	United States	Observational study	Red meat; Processed	Cancer	Negative	Non-industry		No	Non-industry			Negative	Negative/neutral	Non-industry	No	No	No	No	80
Inclusion of red meat in healthful dietary patterns	S. H. McNeill	2014	Other: Meat Science	Meat Science	United States	Not applicable (e.g. no	Review	Red meat	General health	Positive	Industry	National Cattlemen's	Undeclared	Undeclared	National Cattlemen's Beef		Positive	Positive	Industry	Yes	Undeclared	Undeclared	Yes	
Dietary Protein Intake Is Protective Against Loss of Grip Strength Among Older Adults in the Framingham Offspring Cohort	R. R. McLean, K. M. Mangano, M. T. Hannan, D. P. Kiel and S. Sahni	2016	Medicine broadly	J Gerontol A Biol Sci Med Sci	United States	United States	Observational study	Animal protein	Other: Grip strength	Positive	Non-industry		Undeclared	Industry	General Mills Bell Institute of Health	Undeclared	Positive	Positive	Non-industry	No	Undeclared	No	Unclear/Undeclared	80
Meat consumption and pancreatic cancer risk among men and women in the Cancer Prevention Study-II Nutrition Cohort	M. L. McCullough, E. J. Jacobs, R. Shah, P. T. Campbell, Y. Wang, T. J. Hartman and	2017	Disease specific	Cancer Causes Control	United States	United States	Observational study	All meat	Cancer	Neutral/mixed	Non-industry		No	Undeclared			Neutral/mixed	Negative/neutral	Non-industry	No	No	Undeclared	Unclear/Undeclared	100
Partial substitution of red or processed meat with plant-based foods and the risk of type 2 diabetes	M. Maukonen, K. Harald, N. E. Kaartinen, H. Tapanainen, D. Albanes, J. Eriksson, et	2023	Other: Science	Sci Rep	Finland	Finland	Observational study	Red meat; Processed	Diabetes	Neutral/mixed	Non-industry		No	Non-industry			Neutral/mixed	Negative/neutral	Non-industry	No	No	No	No	100
Epidemiologic Burden of Red and Processed Meat Intake on Colorectal Cancer Mortality	C. Mattiuzzi and G. Lippi	2021	Nutrition	Nutr Cancer	Italy	Global	Observational study	Red meat; Processed	Cancer	Negative	Non-industry		No	Non-industry			Negative	Negative/neutral	Non-industry	No	No	No	No	
The Association of Meat Intake With All-Cause Mortality and Acute Myocardial Infarction Is Age-Dependent in Patients With	Ã… O. Matre, A. Van Parys, T. Olsen, T. R. Haugsgjerd, C. M. Baravelli, O. NygÃ¥rd,	2021	Nutrition	Front Nutr	Norway	Norway	Observational study	All meat	Cardiovascular disease; Mortality	Negative	Non-industry		No	Undeclared			Negative	Negative/neutral	Non-industry	No	No	Undeclared	Unclear/Undeclared	60
Association between dietary protein intake and changes in health-related quality of life in older adults: findings from the AusDiab	A. P. Matison, C. M. Milte, J. E. Shaw, D. J. Magliano, R. M. Daly and S. J. Torres	2022	Other: Geriatrics	BMC Geriatr	Australia	Australia	Observational study	Animal protein	General health; Other: Health related quality	Negative	Non-industry		Yes	Non-industry		Not involved in either	Negative	Negative/neutral	Non-industry	No	Yes	No	Yes	60
Maternal protein intake during pregnancy and offspring overweight 20 y later	E. Maslova, D. Rytter, B. H. Bech, T. B. Henriksen, M. A. Rasmussen, S. F. Olsen and	2014	Nutrition	American Journal of Clinical Nutrition	Denmark	Denmark	Observational study	Animal protein	Obesity	Negative	Non-industry		No	Non-industry			Negative	Negative/neutral	Non-industry	No	No	No	No	40
Meat Consumption and Risk of Developing Type 2 Diabetes in the SUN Project: A Highly Educated Middle-Class Population	A. Mari-Sanchis, A. Gea, F. J. Basterra-Gortari, M. A. Martinez-Gonzalez, J. J. Beunza	2016	Medicine broadly	PLoS One	Spain	Spain	Observational study	All meat	Diabetes	Negative	Non-industry		No	Non-industry			Negative	Negative/neutral	Non-industry	No	No	No	No	80
Association between pre-pregnancy consumption of meat, iron intake, and the risk of gestational diabetes: the SUN project	A. MarÃ-Sanchis, G. DÃaz-Jurado, F. J. Basterra-Gortari, C. de la Fuente-Arrillaga,	2017	Nutrition	European Journal of Nutrition	Spain	Spain	Observational study	All meat	Diabetes	Negative	Non-industry		No	Non-industry			Negative	Negative/neutral	Non-industry	No	No	No	No	100
Association between dietary protein intake and prostate cancer risk: evidence from a meta-analysis	Y. Mao, Y. Tie and J. Du	2018	Disease specific	World J Surg Oncol	China	China	Meta analysis	Animal protein	Cancer	Neutral/mixed	Non-industry		No	Undeclared			Neutral/mixed	Negative/neutral	Non-industry	No	No	Undeclared	Unclear/Undeclared	100
Dietary Protein Intake and Risk of Type 2 Diabetes in US Men and Women	V. S. Malik, Y. Li, D. K. Tobias, A. Pan and F. B. Hu	2016	Other: Epidemiology	Am J Epidemiol	United States	United States	Observational study	Animal protein	Diabetes	Negative	Non-industry		No	Non-industry			Negative	Negative/neutral	Non-industry	No	No	No	No	100
Unprocessed red meat in the dietary treatment of obesity: a randomized controlled trial of beef supplementation during weight	F. Magkos, S. I. Rasmussen, M. F. Hjorth, S. Asping, M. I. Rosenkrans, A. M. SjÃ¶din,	2022	Nutrition	American Journal of Clinical Nutrition	Denmark	Denmark	Experimental study	Red meat	Obesity; Cardiovascular disease	Positive	Non-industry		Yes	Industry	Beef Checkoff, National Cattlemen's	Not involved in either	Positive	Positive	Non-industry	No	Yes	Yes	Yes	60
Can Meat and Meat-Products Induce Oxidative Stress?	A. Macho-GonzÃ¡lez, A. GarcimartÃn, M. E. LÃ³pez-Oliva, S. Bastida, J. BenedÃ, G.	2020	Other: Antioxidants	Antioxidants (Basel)	Spain	Not applicable (e.g. no	Review	All meat	Other: Oxidative stress	Neutral/mixed	Non-industry		No	Non-industry			Neutral/mixed	Negative/neutral	Non-industry	No	No	No	No	
Meat intake and risk of hepatocellular carcinoma in two large US prospective cohorts of women and men	Y. Ma, W. Yang, T. Li, Y. Liu, T. G. Simon, J. Sui, et al.	2019	Other: Epidemiology	Int J Epidemiol	China	United States	Observational study	All meat	Cancer	Neutral/mixed	Non-industry		No	Non-industry			Neutral/mixed	Negative/neutral	Non-industry	No	No	No	No	100
Red Meat Consumption and Cancer Risk: A Systematic Analysis of Global Data	H. Ma and X. Qi	2023	Other: Food	Foods	China	Global	Observational study	Red meat	Cancer	Negative	Non-industry		No	Non-industry			Negative	Negative/neutral	Non-industry	No	No	No	No	60
Consumption of meat, traditional and modern processed meat and colorectal cancer risk among the Moroccan population: A large-	S. Deoula, M.; El Kinany, K.; Huybrechts, I.; Gunter, M. J.; Hatime, Z.; Boudouaya, H.	2020	Disease specific	Int J Cancer	Morocco	Morocco	Observational study	All meat	Cancer	Neutral/mixed	Non-industry		No	Non-industry			Neutral/mixed	Negative/neutral	Non-industry	No	No	No	No	100
Assessment of Vascular Function in Response to High-Fat and Low-Fat Ground Beef Consumption in Men	J. R. Lytle, S. T. Stanelle, S. E. Martin, S. B. Smith, D. R. Smith and S. F. Crouse	2023	Nutrition	Nutrients	United States	United States	Experimental study	Beef	Cardiovascular disease	Positive	Unclear/Undeclar		No	Industry	Beef Checkoff	Not involved in either	Positive	Positive	Unclear/Undeclared	Unclear/Undeclar	No	Yes	Yes	0
Dietary meat and protection against sarcopenia	G. S. Lynch and R. Koopman	2018	Other: Meat Science	Meat Science	Australia	Not applicable (e.g. no	Review	All meat	Muscle mass; Other: Sarcopenia	Neutral/mixed	Non-industry		Undeclared	Non-industry			Neutral/mixed	Negative/neutral	Non-industry	No	Undeclared	No	Unclear/Undeclared	
White Meat Consumption, All-Cause Mortality, and Cardiovascular Events: A Meta-Analysis of Prospective Cohort Studies	R. Lupoli, M. Vitale, I. Calabrese, A. Giosue, G. Riccardi and O. Vaccaro	2021	Nutrition	Nutrients	Italy	Global	Meta analysis	White meat	Cardiovascular disease; Mortality	Positive	Non-industry		No	Industry	Barilla Center for Food and Nutrition	Not involved in either	Positive	Positive	Non-industry	No	No	No	No	100
Dietary protein in early pregnancy and gestational diabetes mellitus: a prospective cohort study	T. Luo, H. Chen, H. Wei, Y. Yang, F. Wei and W. Chen	2023	Other: Endocrine	Endocrine	China	China	Observational study	Animal protein	Diabetes	Neutral/mixed	Non-industry		No	Non-industry			Neutral/mixed	Negative/neutral	Non-industry	No	No	No	No	80
Different forms and sources of iron in relation to colorectal cancer risk: a case-control study in China	H. Luo, N. Q. Zhang, J. Huang, X. Zhang, X. L. Feng, Z. Z. Pan, et al.	2019	Nutrition	Br J Nutr	China	China	Observational study	All meat	Cancer	Negative	Non-industry		No	Non-industry			Negative	Negative/neutral	Non-industry	No	No	No	No	80
Red meat consumption and metabolic syndrome in the Costa Rica Heart Study	D. Luan, D. Wang, H. Campos and A. Baylin	2019	Nutrition	European Journal of Nutrition	United States	Costa Rica	Observational study	Red meat	Other: Metabolic syndrome	Negative	Non-industry		No	Non-industry			Negative	Negative/neutral	Non-industry	No	No	No	No	100

Processed Meat Consumption and the Risk of Cancer: A Critical Evaluation of the Constraints of Current Evidence from	M. N. HÃ¤ndel, J. F. Rohde, R. Jacobsen and B. L. Heitmann	2021	Nutrition	Nutrients	Denmark	Not applicable (e.g. no	Review	Processed meat
Processed meat intake and chronic disease morbidity and mortality: An overview of systematic reviews and meta-analyses	M. N. HÃ¤ndel, I. Cardoso, K. M. Rasmussen, J. F. Rohde, R. Jacobsen, S. M. Nielsen,	2019	Medicine broadly	PLoS One	Denmark	Not applicable (e.g. no	Review	Processed meat
Reduction of Red and Processed Meat Intake and Cancer Mortality and Incidence: A Systematic Review and Meta-analysis of	M. A. Han, D. Zeraatkar, G. H. Guyatt, R. W. M. Vernooij, R. El Dib, Y. Zhang, et al.	2019	Medicine broadly	Ann Intern Med	South Korea	Global	Systematic review	Red meat; Processed
Differences in all-cause mortality risk associated with animal and plant dietary protein sources consumption	F. Haghighatdoost, N. Mohammadifard, P. Zakeri, J. Najafian, M. Sadeghi, H.	2023	Other: Science	Sci Rep	Iran	Iran	Observational study	Animal protein
A moderate-carbohydrate diet with plant protein is inversely associated with cardiovascular risk factors: the Korea National Health	K. Ha, K. Nam and Y. Song	2020	Nutrition	Nutrition Journal	South Korea	South Korea	Observational study	Animal protein
Increased Protein Consumption during the Day from an Energy-Restricted Diet Augments Satiety but Does Not Reduce Daily Fat or	J. A. Gwin, K. C. Maki and H. J. Leidy	2017	Nutrition	J Nutr	United States	United States	Experimental study	Beef; Animal protein
Red and processed meat intake and risk of breast cancer: a meta-analysis of prospective studies	J. Guo, W. Wei and L. Zhan	2015	Disease specific	Breast Cancer Res Treat	China	Global	Meta analysis	Red meat; Processed
Meta-Analysis of Randomized Controlled Trials of Red Meat Consumption in Comparison With Various Comparison Diets on	M. Guasch-FerrÃ©, A. Satija, S. A. Blondin, M. Janiszewski, E. Emlen, L. E. O'Connor,	2019	Disease specific	Circulation	United States	Global	Meta analysis	Red meat
Red meat intake and risk of type 2 diabetes in a prospective cohort study of United States females and males	X. Gu, J. P. Drouin-Chartier, F. M. Sacks, F. B. Hu, B. Rosner and W. C. Willett	2023	Nutrition	American Journal of Clinical Nutrition	United States	United States	Observational study	Red meat
Cancer incidence attributable to red and processed meat consumption in Alberta in 2012	A. Grundy, A. E. Poirier, F. Khandwala, A. McFadden, C. M. Friedenreich and D. R.	2016	Medicine broadly	CMAJ Open	Canada	Canada	Observational study	Red meat; Processed
Total, red and processed meat consumption and human health: an umbrella review of observational studies	G. Grosso, S. La Vignera, R. A. Condorelli, J. Godos, S. Marventano, M. Tieri, et al.	2022	Nutrition	Int J Food Sci Nutr		Global	Systematic review	All meat
Dietary Patterns Characterized by High Meat Consumption Are Associated with Other Unhealthy Life Styles and Depression	M. J. GregÃ³rio, A. M. Rodrigues, M. EusÃ©bio, R. D. Sousa, S. Dias, B. AndrÃ©, et	2017	Nutrition	Front Nutr	Portugal	Portugal	Observational study	All meat
Comparison of the Declared Nutrient Content of Plant-Based Meat Substitutes and Corresponding Meat Products and Sausages in	C. GrÃ©a, A. Dittmann, D. Wolff, R. Werner, C. Turban, S. Roser, et al.	2023	Nutrition	Nutrients	Germany	Germany	Observational study	All meat; Other: Meat
Red and processed meat consumption and risk of incident cardiovascular disease and mortality: Isfahan cohort study	N. Grau, N. Mohammadifard, R. Hassannejhad, F. Haghighatdoost, M. Sadeghi, M.	2022	Nutrition	Int J Food Sci Nutr	Iran	Iran	Observational study	Red meat; Processed
Characteristics and Health Benefit of Highly Marbled Wagyu and Hanwoo Beef	T. Gotoh and S. T. Joo	2016	Other: Food Science fo	Korean J Food Sci Anim Resour	South Korea	Not applicable (e.g. no	Review	Beef
Modelling the replacement of red and processed meat with plant-based alternatives and the estimated effect on insulin sensitivity	J. P. Goode, K. J. Smith, M. Breslin, M. Kilpatrick, T. Dwyer, A. J. Venn and C. G.	2023	Nutrition	Br J Nutr	Australia	Australia	Other: Modeling Study	Red meat; Processed
Carcinogenicity of High Consumption of Meat and Lung Cancer Risk Among Non-Smokers: A Comprehensive Meta-Analysis	P. Gnagnarella, S. Caini, P. Maisonneuve and S. Gandini	2018	Nutrition	Nutr Cancer	Italy	Global	Meta analysis	All meat
Benefits and Risks Associated with Meat Consumption during Key Life Processes and in Relation to the Risk of Chronic Diseases	C. Giromini and D. I. Givens	2022	Other: Food	Foods	Italy	Not applicable (e.g. no	Review	All meat
Vegetarianism, low meat consumption and the risk of lung, postmenopausal breast and prostate cancer in a population-based	A. M. Gilsing, M. P. Weijenberg, R. A. Goldbohm, P. C. Dagnelie, P. A. van den	2016	Nutrition	Eur J Clin Nutr	Netherlands	Netherlands	Observational study	All meat
Vegetarianism, low meat consumption and the risk of colorectal cancer in a population based cohort study	A. M. J. Gilsing, L. J. Schouten, R. A. Goldbohm, P. C. Dagnelie, P. A. Van Den Brandt	2015	Other: Science	Sci Rep	Netherlands	Netherlands	Observational study	All meat
The Consumption of Red and Processed Meat and The Risk of Colorectal Cancer: A Case-Control Study among the Tunisian	I. Gharbi, F. Letaief, Z. Dadaa, Y. Yahyaoui, A. Gabsi, H. Maghrebi, et al.	2020	Medicine broadly	Tunis Med	Tunisia	Tunisia	Observational study	Red meat; Processed
Meat and Human Health-Current Knowledge and Research Gaps	N. R. W. Geiker, H. C. Bertram, H. Mejborn, L. O. Dragsted, L. Kristensen, J. R.	2021	Other: Food	Foods	Denmark	Not applicable (e.g. no	Review	All meat
Vegetable but not animal protein intake is associated to a better physical performance: a study on a general population sample of	D. Gazzani, F. Zamboni, F. Spelta, P. Ferrari, V. Mattioli, L. Cazzoletti, et al.	2019	Nutrition	Food & Nutrition Research	Italy	Italy	Observational study	Animal protein
Plasma Metabolite Profiles Associated with the Amount and Source of Meat and Fish Consumption and the Risk of Type 2 Diabetes	J. GarcÃ-a-GavilÃ¡n, S. K. Nishi, I. Paz-Graniel, M. Guasch-FerrÃ©, C. Razquin, C. B.	2022	Nutrition	Mol Nutr Food Res	Spain	Spain	Observational study	All meat
Protein intake from different sources and cognitive decline over 9 years in community-dwelling older adults	R. Gao, Z. Yang, W. Yan, W. Du, Y. Zhou and F. Zhu	2022	Medicine broadly	Frontiers in Public Health	China	China	Observational study	Animal protein
The Association of Fried Meat Consumption With the Gut Microbiota and Fecal Metabolites and Its Impact on Glucose	J. Gao, X. Guo, W. Wei, R. Li, K. Hu, X. Liu, et al.	2021	Disease specific	Diabetes Care	China	China	Experimental study	Other: Fried meat
Prepregnancy Protein Source and BCAA Intake Are Associated with Gestational Diabetes Mellitus in the CARDIA Study	M. D. Gadgil, K. H. Ingram, D. Appiah, J. Rudd, K. M. Whitaker, W. L. Bennett, et al.	2022	Medicine broadly	Int J Environ Res Public Health	United States	United States	Observational study	Animal protein
Measurement of Multiple Vitamin K Forms in Processed and Fresh-Cut Pork Products in the U.S. Food Supply	X. Fu, X. Shen, E. G. Finnan, D. B. Haytowitz and S. L. Booth	2016	Other: Agriculture and Food	J Agric Food Chem	United States	United States	Experimental study	Pork
Processed Meat, but Not Unprocessed Red Meat, Is Inversely Associated with Leukocyte Telomere Length in the Strong Heart	A. M. Fretts, B. V. Howard, D. S. Siscovick, L. G. Best, S. A. A. Beresford, M. Mete, et	2016	Nutrition	J Nutr	United States	United States	Observational study	Red meat; Processed
Consumption of meat is associated with higher fasting glucose and insulin concentrations regardless of glucose and insulin genetic	A. M. Fretts, J. L. Follis, J. A. Nettleton, R. N. Lemaitre, J. S. Ngwa, M. K. Wojczynski,	2015	Nutrition	American Journal of Clinical Nutrition	United States	Global	Meta analysis	All meat
The association of animal and plant protein with successful ageing: a combined analysis of MEDIS and ATTICA epidemiological	A. Foscolou, E. Critselis, S. Tyrovolas, C. Chrysohoou, N. Naumovski, L. S. Sidossis, et	2021	Nutrition	Public Health Nutrition	Greece	Greece	Observational study	Animal protein
Effects of a Multi-component, Resistance-Based Exercise Program Combined with Additional Lean Red Meat on Health-Related	M. B. Formica, J. Gianoudis, C. A. Nowson, S. L. Oâ€™Connell, C. Milte, K. A. Ellis	2023	Nutrition	J Nutr Health Aging	Australia	Australia	Experimental study	Red meat
The importance of protein sources to support muscle anabolism in cancer: An expert group opinion	K. L. Ford, J. Arends, P. J. Atherton, M. Engelen, T. J. M. GonÃ§alves, A. Laviano, et	2022	Nutrition	Clin Nutr	Canada	Not applicable (e.g. no	Other: Opinion paper	Animal protein
The influence of whole grain products and red meat on intestinal microbiota composition in normal weight adults: a randomized	J. Foerster, G. Maskarinec, N. Reichardt, A. Tett, A. Narbad, M. Blaut and H. Boeing	2014	Medicine broadly	PLoS One	Germany	Germany	Experimental study	Red meat
Effect of varying quantities of lean beef as part of a Mediterranean-style dietary pattern on lipids and lipoproteins: a randomized	J. A. Fleming, P. M. Kris-Etherton, K. S. Petersen and D. J. Baer	2021	Nutrition	American Journal of Clinical Nutrition	United States	United States	Experimental study	Beef
Meat intake and risk of gastric cancer in the Stomach cancer Pooling (StoP) project	A. Ferro, V. Rosato, M. Rota, A. R. Costa, S. Morais, C. Pelucchi, et al.	2021	Disease specific	Int J Cancer	Portugal	Global	Meta analysis	Red meat; White
Intake of processed meat, but not sodium, is associated with risk of colorectal cancer: Evidence from a large prospective cohort	Q. Feng, S. H. Wong, J. Zheng, Q. Yang, J. J. Sung and K. K. Tsoi	2021	Nutrition	Clin Nutr	China	UK	Observational study	Processed meat
Consumption of red and processed meat and breast cancer incidence: A systematic review and meta-analysis of prospective	M. S. Farvid, M. C. Stern, T. Norat, S. Sasazuki, P. Vineis, M. P. Weijenberg, et al.	2018	Disease specific	Int J Cancer	United States	Global	Systematic review	Red meat; Processed
Consumption of red meat and processed meat and cancer incidence: a systematic review and meta-analysis of prospective studies	M. S. Farvid, E. Sidahmed, N. D. Spence, K. Mante Angua, B. A. Rosner and J. B.	2021	Other: Epidemiology	Eur J Epidemiol	United States	Global	Systematic review	Red meat; Processed
Adolescent meat intake and breast cancer risk	M. S. Farvid, E. Cho, W. Y. Chen, A. H. Eliassen and W. C. Willett	2015	Disease specific	Int J Cancer	United States	United States	Observational study	Red meat
Dietary protein sources in early adulthood and breast cancer incidence: prospective cohort study	M. S. Farvid, E. Cho, W. Y. Chen, A. H. Eliassen and W. C. Willett	2014	Medicine broadly	BMJ	United States	United States	Observational study	Poultry; Red meat;
The nutritional impact of replacing dietary meat with meat alternatives in the UK: a modelling analysis using nationally	D. N. Farsi, D. Uthumange, J. Munoz Munoz and D. M. Commane	2022	Nutrition	Br J Nutr	UK	UK	Other: Modeling study	All meat; Other: Meat
Dietary Protein Consumption and the Risk of Type 2 Diabetes: ADose-Response Meta-Analysis of Prospective Studies	M. Fan, Y. Li, C. Wang, Z. Mao, W. Zhou, L. Zhang, et al.	2019	Nutrition	Nutrients	China	Global	Meta analysis	Animal protein
Exploration of Metabolic Biomarkers Linking Red Meat Consumption to Ischemic Heart Disease Mortality in the UK Biobank	B. Fan, X. Huang and J. V. Zhao	2023	Nutrition	Nutrients	China	UK	Observational study	Red meat
Plant-Based Diet: Is It as Good as an Animal-Based Diet When It Comes to Protein?	M. W. Ewy, A. Patel, M. G. Abdelmagid, O. Mohamed Elfadil, S. L. Bonnes, B. R.	2022	Nutrition	Current Nutrition Reports	United States	Not applicable (e.g. no	Review	Animal protein
Mortality from different causes associated with meat, heme iron, nitrates, and nitrites in the NIH-AARP Diet and Health Study:	A. Etemadi, R. Sinha, M. H. Ward, B. I. Graubard, M. Inoue-Choi, S. M. Dawsey and	2017	Medicine broadly	BMJ	United States	United States	Observational study	All meat
Red meat and dietary iron intakes are associated with some components of metabolic syndrome: Tehran Lipid and Glucose Study	Z. Esfandiar, F. Hosseini-Esfahani, P. Mirmiran, A. S. Habibi-Moeini and F. Azizi	2019	Medicine broadly	J Transl Med	Iran	Iran	Observational study	Red meat
Replacing red and processed meat with lean or fatty fish and all-cause and cause-specific mortality in Norwegian women. The	T. M. Enget Jensen, T. Braaten, B. K. Jacobsen, D. B. Ibsen and G. Skeie	2023	Nutrition	Br J Nutr	Norway	Norway	Observational study	Red meat; Processed
Red meat, diseases, and healthy alternatives: A critical review	C. Ekmekcioglu, P. Wallner, M. Kundi, U. Weisz, W. Haas and H. P. Hutter	2018	Nutrition	Crit Rev Food Sci Nutr	Austria	Not applicable (e.g. no	Review	Red meat
Effects of different sources of dietary protein on markers of kidney function in individuals with diabetes: a systematic review and	I. Eckert, I. C. Koehler, J. Bauer, F. M. Busnello and F. M. Silva	2022	Nutrition	Nutrition Reviews	Brazil	Global	Systematic review	Animal protein
Dietary source of saturated fat and percentage body fat of patients with type 2 diabetes mellitus: A cross-sectional study	C. K. Duarte, A. L. T. dos Santos, C. Kirst, G. D. S. Nunes, K. de Franceschi, M. J. de	2019	Nutrition	Food Sci Nutr	Brazil	Other	Observational study	Red meat; Processed
Red meat, poultry and fish consumption and risk of diabetes: a 9 year prospective cohort study of the China Kadoorie Biobank	H. Du, Y. Guo, D. A. Bennett, F. Bragg, Z. Bian, M. Chadni, et al.	2020	Disease specific	Diabetologia	UK	China	Observational study	Poultry; Red meat
Unprocessed Red Meat and Processed Meat Consumption, Plasma Metabolome, and Risk of Ischemic Heart Disease: A Prospective	X. Dong, Z. Zhuang, Y. Zhao, Z. Song, W. Xiao, W. Wang, et al.	2023	Medicine broadly	J Am Heart Assoc	China	UK	Observational study	Red meat; Processed
Meat Intake Is Associated with a Higher Risk of Ulcerative Colitis in a Large European Prospective Cohort StudyÃ¸	C. Dong, S. S. M. Chan, P. Jantchou, A. Racine, B. Oldenburg, E. Weiderpass, et al.	2022	Disease specific	J Crohns Colitis	France	Europe	Observational study	All meat
Should we recommend reductions in saturated fat intake or in red/processed meat consumption? The SUN prospective cohort	L. J. Dominguez, M. Bes-Rastrollo, F. J. Basterra-Gortari, A. Gea, M. Barbagallo and	2018	Nutrition	Clin Nutr	Italy	Spain	Observational study	Red meat; Processed
Carcinogenicity of consumption of red meat and processed meat: A review of scientific news since the IARC decision	J. L. Domingo and M. Nadal	2017	Other: Food and Chemical	Food and Chemical Toxicology	Spain	Not applicable (e.g. no	Review	Red meat; Processed
Meat and mental health: a systematic review of meat abstention and depression, anxiety, and related phenomena	U. Dobersek, G. Wy, J. Adkins, S. Altmeyer, K. Krout, C. J. Lavie and E. Archer	2021	Nutrition	Crit Rev Food Sci Nutr	United States	Global	Systematic review	All meat
Meat and mental health: A meta-analysis of meat consumption, depression, and anxiety	U. Dobersek, K. Teel, S. Altmeyer, J. Adkins, G. Wy and J. Peak	2021	Nutrition	Crit Rev Food Sci Nutr	United States	Global	Meta analysis	All meat
Red meat consumption and its association with hypertension and hyperlipidaemia among adult Maasai pastoralists of Ngorongoro	E. J. Diarz, B. J. Leyaro, S. L. Kivuyo, B. J. Ngowi, S. E. Msuya, S. G. Mfinanga, et al.	2020	Medicine broadly	PLoS One	Tanzania	Tanzania	Observational study	Red meat
The association between meat and fish consumption and bladder cancer risk: a pooled analysis of 11 cohort studies	M. Dianatinasab, A. Wesselius, T. de Loeij, A. Salehi-Abargouei, E. Y. W. Yu, M.	2021	Other: Epidemiology	Eur J Epidemiol	Netherlands	Global	Meta analysis	All meat
Red and processed meat intake and cancer risk: Results from the prospective NutriNet-SantÃ© cohort study	A. Diallo, M. Deschasaux, P. Latino-Martel, S. Hercberg, P. Galan, P. Fassier, et al.	2017	Disease specific	Int J Cancer	France	France	Observational study	Red meat; Processed
Association of meat consumption with the risk of gastrointestinal cancers: a systematic review and meta-analysis	Y. Di, L. Ding, L. Gao and H. Huang	2023	Disease specific	BMC Cancer	China	Global	Systematic review	All meat
Effects of Animal and Vegetable Proteins on Gut Microbiota in Subjects with Overweight or Obesity	C. Di Rosa, L. Di Francesco, C. Spiezia and Y. M. Khazrai	2023	Nutrition	Nutrients	Italy	Not applicable (e.g. no	Review	Animal protein
Associations between Red Meat Intakes and the Micronutrient Intake and Status of UK Females: A Secondary Analysis of the UK	E. Derbyshire	2017	Nutrition	Nutrients	UK	UK	Observational study	Red meat
Meat consumption and risk of squamous cell carcinoma of the lung: a case-control study in Uruguayan men	H. Deneo-Pellegrini, A. L. Ronco and E. De Stefani	2015	Nutrition	Nutr Cancer	Uruguay	Uruguay	Observational study	All meat
Population attributable fractions for colorectal cancer and red and processed meats in Colombia - a macro-simulation study	E. De Vries, D. C. Quintero, G. HenrÃ-quez-Mendoza and O. F. HerrÃ¡n	2017	Medicine broadly	Colomb Med (Cali)	Colombia	Colombia	Other: Modeling study	Red meat; Processed
Processed meat consumption and squamous cell carcinoma of the oesophagus in a large case-control study in Uruguay	E. de Stefani, P. Boffetta, A. L. Ronco, H. Deneo-Pellegrini, P. Correa, G. Acosta and	2014	Disease specific	Asian Pac J Cancer Prev	Uruguay	Uruguay	Observational study	Processed meat
Estimation of the burden of disease attributable to red meat consumption in France: Influence on colorectal cancer and	J. De Oliveira Mota, G. BouÃ©, S. Guillou, F. Pierre and J. M. MembrÃ©	2019	Other: Food and Chemical	Food and Chemical Toxicology	France	France	Other: Modeling study	Red meat
Associations of the consumption of unprocessed red meat and processed meat with the incidence of cardiovascular disease and	G. de Medeiros, G. X. B. Mesquita, S. Lima, D. F. O. Silva, K. P. M. de Azevedo, I.	2023	Nutrition	Crit Rev Food Sci Nutr	Brazil	Global	Systematic review	Red meat; Processed
	J. de Batlle, E. Gracia-Lavedan, D. Romaguera, M. Mendez, G. CastaÃ±o-Vinyals, V.	2018	Nutrition	European Journal of Nutrition	France	Spain	Observational study	All meat
Consumption Patterns and the Nutritional Contribution of Total, Processed, Fresh, and Fresh-Lean Pork to the U.S. Diet	L. Y. Datlow, M. Leventhal, J. King and T. C. Wallace	2023	Nutrition	Nutrients	United States	United States	Observational study	Pork; Processed meat
Associations between dietary intake of total protein and sources of protein (plant vs. animal) and risk of all-cause and cause-	A. Das, R. Cumming, V. Naganathan, F. Blyth, D. G. L. Couteur, D. J. Handelsman, et	2022	Nutrition	J Hum Nutr Diet	Australia	Australia	Observational study	Animal protein
The association of red meat consumption and mental health in women: A cross-sectional study	M. Darooghegi Mofrad, H. Mozaffari, A. Sheikhi, B. Zamani and L. Azadbakht	2021	Medicine broadly	Complement Ther Med	Iran	Iran	Observational study	Red meat
	E. Daneshzad, M. Askari, M. Moradi, S. Ghorabi, T. Rouzitalab, J. Heshmati and L.	2021	Nutrition	Clin Nutr ESPEN	Iran	Global	Systematic review	Red meat
White Meat Consumption and Cardiometabolic Risk Factors: A Review of Recent Prospective Cohort Studies	E. Damigou, R. I. Kosti and D. B. Panagiotakos	2022	Nutrition	Nutrients	Greece	Not applicable (e.g. no	Review	White meat
Associations between meat type consumption pattern and incident cardiovascular disease: The ATTICA epidemiological cohort	E. Damigou, R. I. Kosti, C. Anastasiou, C. Chrysohoou, F. Barkas, P. S. Adamidis, et al.	2023	Other: Meat Science	Meat Science	Greece	Greece	Observational study	All meat
Dietary protein intake and mortality among survivors of liver cirrhosis: a prospective cohort study	Daftari, G.; Tehrani, A. N.; Pashayee-khamene, F.; Karimi, S.; Ahmadzadeh, S.;	2023	Disease specific	BMC Gastroenterol	Iran	Iran	Observational study	Animal protein
Association of white and red meat consumption with general and abdominal obesity: a cross-sectional study among a population	A. Dabbagh-Moghadam, H. Mozaffari-Khosravi, M. Nasiri, A. Miri, M. Rahdar and	2017	Medicine broadly	Eat Weight Disord	Iran	Iran	Observational study	Red meat; White meat
Nutritional Quality of Meat Analogues: Results From the Food Labelling of Italian Products (FLIP) Project	Cutroneo, S.; Angelino, D.; Tedeschi, T.; Pellegrini, N.; Martini, D.	2022	Nutrition	Front Nutr	Italy	Italy	Observational study	All meat; Other: Meat
Red and processed meat consumption and risk of bladder cancer: a dose-response meta-analysis of epidemiological studies	Crippa, A.; Larsson, S. C.; Discacciati, A.; Wolk, A.; Orsini, N.	2018	Nutrition	European Journal of Nutrition	Sweden	Global	Meta analysis	Red meat; Processed
Assessing the effects of alternative plant-based meats v. animal meats on biomarkers of inflammation: a secondary analysis of the	Crimarco, A.; Landry, M. J.; Carter, M. M.; Gardner, C. D.	2022	Nutrition	J Nutr Sci	United States	United States	Experimental study	All meat; Other: Meat
Comparative Assessment of the Nutritional Profile of Meat Products and Their Plant-Based Analogues	Costa-Catala, J.; Toro-Funes, N.; Comas-BastÃ©, O.; HernÃ¡ndez-Macias, S.;	2023	Nutrition	Nutrients	Spain	Spain	Observational study	All meat; Other: Meat
Poultry Consumption and Human Cardiometabolic Health-Related Outcomes: A Narrative Review	Connolly, G.; Campbell, W. W.	2023	Nutrition	Nutrients	United States	Not applicable (e.g. no	Review	Poultry
	Collatuzzo, G.; Etemadi, A.; Sotoudeh, M.; Nikmanesh, A.; Poustchi, H.; Khoshnia,	2022	Disease specific	Int J Cancer	Italy	Iran	Observational study	Red meat; Processed
Meat versus meat alternatives: which is better for the environment and health? A nutritional and environmental analysis of animal	Coffey, A. A.; Lillywhite, R.; Oyebode, O.	2023	Nutrition	J Hum Nutr Diet	UK	UK	Observational study	All meat; Other: Meat
Protein Intake and Frailty in Older Adults: A Systematic Review and Meta-Analysis of Observational Studies	Coelho-Junior, H. J.; Calvani, R.; Picca, A.; Tosato, M.; Landi, F.; Marzetti, E.	2022	Nutrition	Nutrients	Italy	Global	Systematic review	Animal protein
High relative consumption of vegetable protein is associated with faster walking speed in well-functioning older adults	Coelho-Junior, H. J.; Calvani, R.; GonÃ§alves, I. O.; Rodrigues, B.; Picca, A.; Landi, F.;	2019	Other: Aging Clinical and	Aging Clin Exp Res	Italy	Brazil	Observational study	Animal protein
Red but not white meat consumption is associated with metabolic syndrome, insulin resistance and lipid peroxidation in Brazilian	Cocate, P. G.; Natali, A. J.; Oliveira, A. D.; Alfenas, R. D. C. G.; Peluzio, M. D. C. G.;	2020	Disease specific	Eur J Prev Cardiol	Brazil	Brazil	Observational study	Red meat; White meat
High- and normal-protein diets improve body composition and glucose control in adults with type 2 diabetes: a randomized trial	Clina, J. G.; Sayer, R. D.; Pan, Z.; Cohen, C. W.; McDermott, M. T.; Catenacci, V. A.;	2023	Disease specific	Obesity (Silver Spring)	United States	United States	Experimental study	Beef
Linkage between a plant-based diet and age-related eye diseases: a systematic review and meta-analysis	Cirone, C.; Cirone, K. D.; Malvankar-Mehta, M. S.	2023	Nutrition	Nutrition Reviews	Canada	Global	Systematic review	Animal protein
Animal Protein Intake Is Positively Associated with Metabolic Syndrome Risk Factors in Middle-Aged Korean Men	Chung, S.; Chung, M. Y.; Choi, H. K.; Park, J. H.; Hwang, J. T.; Joung, H.	2020	Nutrition	Nutrients	South Korea	South Korea	Observational study	Animal protein
Global red and processed meat trade and non-communicable diseases	Chung, M. G.; Li, Y.; Liu, J.	2021	Medicine broadly	BMJ Glob Health	United States	Global	Review	All meat
Characteristics of pork belly consumption in South Korea and their health implication	Choe, J. H.; Yang, H. S.; Lee, S. H.; Go, G. W.	2015	Veterinary sciences	J Anim Sci Technol	South Korea	Not applicable (e.g. no	Review	Pork
Association of Red Meat Intake and Colorectal Cancer among East-Asians: A Systematic Review and Meta-analysis of Observational	Cheung, H. S.; Lu, L. Y.; So, W. L.; Wong, H. W.; Wong, S. H.; Mak, C. M.	2023	Disease specific	J Gastrointestin Liver Dis	China	China	Systematic review	Red meat
Associations of specific dietary protein with longitudinal insulin resistance, prediabetes and type 2 diabetes: The Rotterdam Study	Chen, Z.; Franco, O. H.; Lamballais, S.; Ikram, M. A.; Schoufour, J. D.; Muka, T.;	2020	Nutrition	Clin Nutr	Netherlands	Netherlands	Observational study	Animal protein
The risk of urinary tract infection in vegetarians and non-vegetarians: a prospective study	Chen, Y. C.; Chang, C. C.; Chiu, T. H. T.; Lin, M. N.; Lin, C. L.	2020	Other: Scientific reports	Sci Rep	Taiwan	Taiwan	Observational study	All meat
Red and Processed Meat Intake, Polygenic Risk Score, and Colorectal Cancer Risk	Chen, X.; Hoffmeister, M.; Brenner, H.	2022	Nutrition	Nutrients	Germany	Germany	Observational study	Red meat; Processed
Beef intake and risk of rheumatoid arthritis: Insights from a cross-sectional study and two-sample Mendelian randomization	Chen, W.; Liu, K.; Huang, L.; Mao, Y.; Wen, C.; Ye, D.; He, Z.	2022	Nutrition	Front Nutr	China	UK	Observational study	Beef
Stroke mortality attributable to high red meat intake in China and South Korea: An age-period-cohort and joinpoint analysis	Chen, F.; Hu, W.; Chen, S.; Si, A.; Zhang, Y.; Ma, J.	2022	Nutrition	Front Nutr	China	China, South Korea	Observational study	Red meat
Long-Time Trend of Colorectal Cancer Mortality Attributable to High Processed Meat Intake in China and a Bayesian Projection	Chen, F.; Chen, S.; Luo, Y.; Si, A.; Yang, Y.; Li, Y.; Hu, W.; Zhang, Y.	2022	Other: Environment and	Int J Environ Res Public Health	China	China	Other: Modeling study	Processed meat
Is vegetarian diet associated with a lower risk of breast cancer in Taiwanese women?	Chang, Y. J.; Chang, Y. J.; Hou, Y. C.; Chen, L. J.; Wu, J. H.; Wu, C. C.; Chung, K. P.	2017	Medicine broadly	BMC Public Health	Taiwan	Taiwan	Observational study	All meat
High Protein Intake Is Associated with Lower Risk of All-Cause Mortality in Community-Dwelling Chinese Older Men and Women	Chan, R.; Leung, J.; Woo, J.	2019	Nutrition	J Nutr Health Aging	China	China	Observational study	Animal protein
A systematic review of the effects of plant compared with animal protein sources on features of metabolic syndrome	Chalvon-Demersay, T.; Azzout-Marniche, D.; Arfsten, J.; Egli, L.; Gaudichon, C.;	2017	Nutrition	J Nutr	France	Global	Systematic review	Animal protein
Association of Meat Subtypes With Colorectal Polyp Prevalence: Finding From the Lanxi Pre-colorectal Cancer Cohort in China	Chai, X.; Li, Y.; Yin, Z.; Wu, F.; Hu, P.; Liu, X.; Tong, S.; Zhuang, P.; Zhang, Y.; Zheng,	2022	Nutrition	Front Nutr	China	China	Observational study	All meat
Dietary Red and Processed Meat Intake and Markers of Adiposity and Inflammation: The Multiethnic Cohort Study	Chai, W.; Morimoto, Y.; Cooney, R. V.; Franke, A. A.; Shvetsov, Y. B.; Le Marchand,	2017	Nutrition	J Am Coll Nutr	United States	United States	Observational study	Red meat; Processed
Dietary sources of N-nitroso compounds and bladder cancer risk: findings from the Los Angeles bladder cancer study	Catsburg, C. E.; Gago-Dominguez, M.; Yuan, J. M.; Castelao, J. E.; Cortessis, V. K.;	2014	Disease specific	Int J Cancer	United States	United States	Observational study	Processed meat
Red meat's role in addressing 'nutrients of public health concern'	Cashman, K. D.; Hayes, A.	2017	Other: Meat Science	Meat Science	Ireland	Not applicable (e.g. no	Review	Red meat
Meat subtypes and their association with colorectal cancer: Systematic review and meta-analysis	Carr, P. R.; Walter, V.; Brenner, H.; Hoffmeister, M.	2016	Disease specific	Int J Cancer	Germany	Not applicable (e.g. no	Review	All meat
Associations of red and processed meat with survival after colorectal cancer and differences according to timing of dietary	Carr, P. R.; Jansen, L.; Walter, V.; Kloor, M.; Roth, W.; Blaker, H.; Chang-Claude, J.;	2016	Nutrition	American Journal of Clinical Nutrition	Germany	Germany	Observational study	Red meat; Processed
Associations of red and processed meat intake with major molecular pathological features of colorectal cancer	Carr, P. R.; Jansen, L.; Bienert, S.; Roth, W.; Herpel, E.; Kloor, M.; BlÃ¤ker, H.; Chang-	2017	Other: Epidemiology	Eur J Epidemiol	Germany	Germany	Observational study	Red meat; Processed
Meat intake and risk of colorectal polyps: results from a large population-based screening study in Germany	Carr, P. R.; Holleczek, B.; Stegmaier, C.; Brenner, H.; Hoffmeister, M.	2017	Nutrition	American Journal of Clinical Nutrition	Germany	Germany	Observational study	All meat
Association Between Intake of Red and Processed Meat and Survival in Patients With Colorectal Cancer in a Pooled Analysis	Carr, P. R.; Banbury, B. L.; Berndt, S. I.; Campbell, P. T.; Chang-Claude, J.; Hayes, R.	2019	Disease specific	Clin Gastroenterol Hepatol	Germany	Global	Meta analysis	Red meat; Processed
A review and meta-analysis of prospective studies of red and processed meat, meat cooking methods, heme iron, heterocyclic	Bylsma, L. C.; Alexander, D. D.	2015	Nutrition	Nutrition Journal	United States	Not applicable (e.g. no	Review	Red meat; Processed
Association of Animal and Plant Protein Intake with All-Cause and Cause-Specific Mortality in a Japanese Cohort	Budhathoki, S.; Sawada, N.; Iwasaki, M.; Yamaji, T.; Goto, A.; Kotemori, A.; Ishihara,	2019	Medicine broadly	JAMA Intern Med	Japan	Japan	Observational study	Animal protein
A Contemporary Review of the Relationship between Red Meat Consumption and Cardiovascular Risk	S. Bronzato and A. Durante	2017	Medicine broadly	Int J Prev Med	Italy	Not applicable (e.g. no	Review	Red meat
High-Protein Foods and Physical Activity Protect Against Age-Related Muscle Loss and Functional Decline	M. L. Bradlee, J. Mustafa, M. R. Singer and L. L. Moore	2017	Medicine broadly	J Gerontol A Biol Sci Med Sci	United States	United States	Observational study	Animal protein
The impact of red and processed meat consumption on cardiovascular disease risk in women	S. Bovalino, G. Charleson and C. Szoeke	2015	Nutrition	Nutrition	Australia	Australia	Observational study	Red meat; Processed
Long-term intake of animal flesh and risk of developing hypertension in three prospective cohort studies	L. Borgi, G. C. Curhan, W. C. Willett, F. B. Hu, A. Satija and J. P. Forman	2015	Disease specific	J Hypertens	United States	United States	Observational study	All meat
Meat Intake, Cooking Methods, Doneness Preferences and Risk of Gastric Adenocarcinoma in the MCC-Spain Study	E. Boldo, N. FernÃ¡ndez de Larrea, M. PollÃ¡n, V. MartÃ-n, M. ObÃ³n-Santacana, M.	2022	Nutrition	Nutrients	Spain	Spain	Observational study	All meat
Meat intake, methods and degrees of cooking and breast cancer risk in the MCC-Spain study	E. Boldo, A. CastellÃ³, N. AragonÃ©s, P. Amiano, B. PÃ©rez-GÃ³mez, G. CastaÃ±o-	2018	Other: Women's health	Maturitas	Spain	Spain	Observational study	All meat
The impact of red and processed meat consumption on cancer and other health outcomes: Epidemiological evidences	L. D. Boada, L. A. HenrÃ-quez-HernÃ¡ndez and O. P. Luzardo	2016	Other: Food and chemical	Food and Chemical Toxicology	Spain	Not applicable (e.g. no	Review	Red meat; Processed
A Higher Mediterranean Diet Score, Including Unprocessed Red Meat, Is Associated with Reduced Risk of Central Nervous System	L. J. Black, K. Baker, A. L. Ponsonby, I. van der Mei, R. M. Lucas and G. Pereira	2019	Nutrition	J Nutr	Australia	Australia	Observational study	Red meat
The Impact of Macronutrient Intake on Sex Steroids During Onset of Puberty	F. M. Biro, S. S. Summer, B. Huang, C. Chen, J. Benoit and S. M. Pinney	2022	Medicine broadly	J Adolesc Health	United States	United States	Observational study	Animal protein
Red meats: time for a paradigm shift in dietary advice	M. A. Binnie, K. Barlow, V. Johnson and C. Harrison	2014	Other: Meat Science	Meat Science	Canada	Not applicable (e.g. no	Review	Red meat; Processed
Anxiety is a potential effect modifier of the association between red and processed meat consumption and cancer risk: findings	M. Beslay, B. Srour, M. Deschasaux, E. FouchÃ©, N. Naud, V. BacquiÃ©, et al.	2021	Nutrition	European Journal of Nutrition	France	France	Observational study	Red meat; Processed
Processed and unprocessed red meat and risk of colorectal cancer: Analysis by tumor location and modification by time	A. M. Bernstein, M. Song, X. Zhang, A. Pan, M. Wang, C. S. Fuchs, et al.	2015	Medicine broadly	PLoS One	United States	United States	Observational study	Red meat
Dietary plant and animal protein intake and decline in estimated glomerular filtration rate among elderly women: a 10-year	A. Bernier-Jean, R. L. Prince, J. R. Lewis, J. C. Craig, J. M. Hodgson, W. H. Lim, et al.	2020	Disease specific	Nephrol Dial Transplant	Australia	Australia	Observational study	Animal protein
Effects of red meat, white meat, and nonmeat protein sources on atherogenic lipoprotein measures in the context of low	N. Bergeron, S. Chiu, P. T. Williams, S M King and R. M. Krauss	2019	Nutrition	American Journal of Clinical Nutrition	United States	United States	Experimental study	Red meat; White meat
Differences in survival associated with processed and with nonprocessed red meat consumption	A. Bellavia, S. Larsson, M. Bottai, A. Wolk, N. Orsini	2014	Medicine broadly	American Journal of Clinical Nutrition	United States	United States	Observational study	Red meat; Processed
Differential acute postprandial effects of processed meat and isocaloric vegan meals on the gastrointestinal hormone response in	L. Belinova, H. Kahleova, H. Malinska, O. Topolcan, J. Vrzalova, O. Oliyarnyk, et al.	2014	Medicine broadly	PLoS One	Czech Republic	Czech Republic	Experimental study	Processed meat
Protein intake, metabolic status and the gut microbiota in different ethnicities: Results from two independent cohorts	P. Bel Lassen, I. Attaye, S. Adriouch, M. Nicolaou, J. Aron-Wisnewsky, T. Nielsen, et	2021	Nutrition	Nutrients	France	Europe	Observational study	Animal protein
Higher meat intake is positively associated with higher risk of developing pancreatic cancer in an age-dependent manner and are	A. J. Beaney, P. J. R. Banim, R. Luben, M. A. H. Lentjes, K. T. Khaw and A. R. Hart	2017	Disease specific	Pancreas	UK	Europe	Observational study	All meat
Protein-Rich Food Ingestion Stimulates Mitochondrial Protein Synthesis in Sedentary Young Adults of Different BMIs	J. W. Beals, R. W. A. Mackenzie, S. Van Vliet, S. K. Skinner, B. A. Pagni, G. M.	2017	Other: Endocrinology and	J Clin Endocrinol Metab	United States	United States	Experimental study	Pork
Health Risks Associated with Meat Consumption: A Review of Epidemiological Studies	E. Battaglia Richi, B. Baumer, B. Conrad, R. Darioli, A. Schmid and U. Keller	2015	Nutrition	International Journal for Vitamin and	Switzerland	Not applicable (e.g. no	Review	All meat
Integrating nutrient bioavailability and co-production links when identifying sustainable diets: How low should we reduce meat	T. BarrÃ©, M. Perignon, R. Gazan, F. Vieux, V. Micard, M. J. Amiot and N. Darmon	2018	Medicine broadly	PLoS One	France	Not applicable (e.g. no	Other: Modeling study	All meat
Meat consumption as a risk factor for type 2 diabetes	N. Barnard, S. Levin and C. Trapp	2014	Nutrition	Nutrients	United States	Not applicable (e.g. no	Review	All meat
Plant-Based Dietary Patterns versus Meat Consumption and Prevalence of Impaired Glucose Intolerance and Diabetes Mellitus: A	C. L. Baleato, J. J. A. Ferguson, C. Oldmeadow, G. D. Mishra and M. L. Garg	2022	Nutrition	Nutrients	Australia	Australia	Observational study	All meat
Effect of Iso-Caloric Substitution of Animal Protein for Other Macro Nutrients on Risk of Overall, Cardiovascular and Cancer	R. Bajracharya, V. Katzke, T. Mukama and R. Kaaks	2023	Nutrition	Nutrients	Germany	Germany	Systematic review	Animal protein
Food Sources of Animal Protein in Relation to Overall and Cause-Specific Mortality-Causal Associations or Confounding? An	R. Bajracharya, R. Kaaks and V. Katzke	2023	Nutrition	Nutrients	Germany	Germany	Observational study	All meat
Red and processed meat consumption and food insecurity are associated with hypertension; Analysis of the National Health and	D. M. Ba, X. Gao, V. M. Chinchilli, D. Liao, J. P. Richie and L. Al-Shaar	2021	Disease specific	J Hypertens	United States	United States	Observational study	Red meat; Processed
Animal-protein intake is associated with insulin resistance in Adventist Health Study 2 (AHS-2) calibration substudy participants: A	B. Azemati, S. Rajaram, K. Jaceldo-Siegl, J. Sabate, D. Shavlik, G. E. Fraser and E. H.	2017	Nutrition	Curr Dev Nutr	United States	United States	Observational study	Animal protein
Red Meat and Colorectal Cancer	N. F. Aykan	2015	Disease specific	Oncol Rev	Turkey	Not applicable (e.g. no	Review	Red meat; Processed
Serum Ferritin Is Associated with Metabolic Syndrome and Red Meat Consumption	F. Avila, G. EcheverrÃ-a, D. PÃ©rez, C. Martinez, P. Strobel, O. Castillo, et al.	2015	Medicine broadly	Oxid Med Cell Longev	Chile	Chile	Observational study	Red meat
The Impact of Meat Intake on Bladder Cancer Incidence: Is It Really a Relevant Risk?	A. Aveta, C. Cacciapuoti, B. Barone, E. Di Zazzo, F. Del Giudice, M. Maggi, et al.	2022	Disease specific	Cancers (Basel)	Italy	Not applicable (e.g. no	Review	All meat
Associations of Total Protein or Animal Protein Intake and Animal Protein Sources with Risk of Kidney Stones: A Systematic Review	F. Asoudeh, S. Talebi, A. Jayedi, W. Marx, M. T. Najafi and H. Mohammadi	2022	Nutrition	Adv Nutr	Iran	Global	Systematic review	Animal protein
Protein intake in children and growth and risk of overweight or obesity: A systematic review and meta-analysis	E. K. Arnesen, B. Thorisdottir, C. Lamberg-Allardt, L. BÃ¤rebring, B. Nwaru, J.	2022	Nutrition	Food & Nutrition Research	Norway	Nordic Countries	Systematic review	Animal protein
Association between processed meat intake and asthma symptoms in the French NutriNet-SantÃ© cohort	R. M. Andrianasolo, S. Hercberg, M. Touvier, N. Druesne-Pecollo, M. Adjibade, E.	2020	Nutrition	European Journal of Nutrition	France	France	Observational study	Processed meat
Red and processed meat consumption and breast cancer: UK Biobank cohort study and meta-analysis	J. J. Anderson, N. D. M. Darwis, D. F. Mackay, C. A. Celis-Morales, D. M. Lyall, N.	2018	Disease specific	Eur J Cancer	UK	UK	Observational study	Red meat; Processed
Intake of Red and Processed Meat, Use of Non-Steroid Anti-Inflammatory Drugs, Genetic Variants and Risk of Colorectal Cancer: A	V. Andersen, U. Halekoh, A. TjÃ¸nneland, U. Vogel and T. I. Kopp	2019	Other: Molecular Sciences	Int J Mol Sci	Denmark	Denmark	Observational study	Red meat; Processed
Red meat intake, NAT2, and risk of colorectal cancer: a pooled analysis of 11 studies	A. N. Ananthakrishnan, M. Du, S. I. Berndt, H. Brenner, B. J. Caan, G. Casey, et al.	2014	Disease specific	Cancer Epidemiol Biomarkers Prev	United States	Global	Meta analysis	Red meat
Fresh- and lean-pork intake in relation to functional limitations among US older adults, 2005-2016	R. An, S. M. Nickols-Richardson, R. J. Alston, S. Shen and C. Clarke	2020	Nutrition	Nutr Health	United States	United States	Observational study	Pork
Fresh and fresh lean beef intake in relation to functional limitations among US older adults, 2005-2016	R. An, S. M. Nickols-Richardson, R. J. Alston and S. Shen	2019	Medicine broadly	Am J Health Behav	United States	United States	Observational study	Beef
Total, Fresh, Lean, and Fresh Lean Beef Consumption in Relation to Nutrient Intakes and Diet Quality among U.S. Adults,	R. An, S. Nickols-Richardson, R. Alston, S. Shen and C. Clarke	2019	Nutrition	Nutrients	United States	United States	Observational study	Beef
Pork Consumption in Relation to Body Weight and Composition: A Systematic Review and Meta-analysis	R. An, J. Liu and R. Liu	2020	Medicine broadly	Am J Health Behav	United States	Global	Systematic review	Pork
The effect of nocturnal "meat" versus "vegetarian" dinners on sleep quality and daily functioning	G. Amit, S. Simona, S. Nohar, M. Niv and D. Yaron	2021	Other: Sleep and Breathing	Sleep Breath	Israel	Israel	Experimental study	All meat
Unprocessed red meat and processed meat consumption and risk of stroke in the Spanish cohort of the European Prospective	P. Amiano, S. Chamosa, N. Etxezarreta, L. Arriola, M. J. SÃ¡nchez, E. Ardanaz, et al.	2016	Nutrition	Eur J Clin Nutr	Spain	Spain	Observational study	Red meat; Processed
A prospective study on total protein, plant protein and animal protein in relation to the risk of incident chronic kidney disease	S. Alvirdizadeh, E. Yuzbashian, P. Mirmiran, S. Eghtesadi and F. Azizi	2020	Other: Nephrology	BMC Nephrol	Iran	Iran	Observational study	Animal protein
Red and Processed Meat and Mortality in a Low Meat Intake Population	S. M. Alshahrani, G. E. Fraser, J. SabatÃ©, R. Knutsen, D. Shavlik, A. Mashchak, et	2019	Nutrition	Nutrients	Saudi Arabia	North America	Observational study	Red meat; Processed
State-of-the-Art Review: Evidence on Red Meat Consumption and Hypertension Outcomes	T. S. Allen, H. S. Bhatia, A. C. Wood, S. R. Momin and M. A. Allison	2022	Disease specific	Am J Hypertens	United States	Not applicable (e.g. no	Review	Red meat
The potential impact of animal protein intake on global and abdominal obesity: evidence from the Observation of Cardiovascular	A. Alkerwi, N. Sauvageot, J. D. Buckley, A. F. Donneau, A. Albert, M. Guillaume, et	2015	Nutrition	Public Health Nutrition	Luxembourg	Luxembourg	Observational study	Animal protein
Vegan, Vegetarian and Meat-Based Diets in Saudi Arabia	N. AlHusseini, M. Sajid, Y. Akkielah, T. Khalil, M. Alatout, P. Cahusac, et al.	2021	Medicine broadly	Cureus	Saudi Arabia	Saudi Arabia	Observational study	All meat
Dietary Protein Sources and Muscle Mass over the Life Course: The Lifelines Cohort Study	N. V. Alexandrov, C. Eelderink, C. M. Singh-Povel, G. J. Navis, S. J. L. Bakker and E.	2018	Nutrition	Nutrients	Netherlands	Netherlands	Observational study	Animal protein
Red Meat and Colorectal Cancer: A Quantitative Update on the State of the Epidemiologic Science	D. D. Alexander, D. L. Weed, P. E. Miller and M. A. Mohamed	2015	Nutrition	J Am Coll Nutr	United States	Global	Meta analysis	Red meat
Red meat intake and risk of coronary heart disease among US men: prospective cohort study	L. Al-Shaar, A. Satija, D. D. Wang, E. B. Rimm, S. A. Smith-Warner, M. J. Stampfer, et	2020	Medicine broadly	BMJ	United States	United States	Observational study	Red meat; Processed
Nutritional Effects of Removing a Serving of Meat or Poultry from Healthy Dietary Patterns-A Dietary Modeling Study	S. Agarwal, K. R. McCullough and V. L. Fulgoni	2023	Nutrition	Nutrients	United States	Not applicable (e.g. no	Other: Modeling Study	All meat

Association of lunch meat consumption with nutrient intake, diet quality and health risk factors in US children and adults: NHANES	S. Agarwal, V. L. Fulgoni and E. P. Berg	2015	Nutrition	Nutrition Journal	United States	United States	Observational study	Other: Lunch meat	Nutritional outcomes; General health	Positive	Industry	NutriScience LLC,	Yes	Industry	North American Meat Institute	Undeclared	Positive	Positive	Industry	Yes	Yes	Yes	Yes	80
Association of Pork (All Pork, Fresh Pork and Processed Pork) Consumption with Nutrient Intakes and Adequacy in US Children (Age	S. Agarwal and V. L. Fulgoni	2023	Nutrition	Nutrients	United States	United States	Observational study	Pork	Nutritional outcomes	Positive	Industry	NutriScience LLC,	Yes	Industry	Pork Checkoff	Not involved in either	Positive	Positive	Industry	Yes	Yes	Yes	Yes	60
Contribution of beef to key nutrient intakes in American adults: an updated analysis with NHANES 2011-2018		2022	Nutrition	Nutr Res	United States	United States	Observational study	Beef	Nutritional outcomes	Positive	Industry	NutriScience LLC,	Yes	Industry	Beef Checkoff	Undeclared	Positive	Positive	Industry	Yes	Yes	Yes	Yes	40
	M. M. Adeva-Andany, E. RaÃ±al-MuÃ-Ã±o, M. Vila-Altesor, C. FernÃ¡ndez-	2019	Nutrition	Clin Nutr ESPEN	Spain	Not applicable (e.g. no	Review	Animal protein	Diabetes	Negative	Non-industry		No	No funding received			Negative	Negative/neutral	Non-industry	No	No	No	No	
The differential effect of animal versus vegetable dietary protein on the clinical manifestations of diabetic kidney disease in	M. M. Adeva-Andany, C. FernÃ¡ndez-FernÃ¡ndez, N. Carneiro-Freire, M. Vila-Altesor	2022	Nutrition	Clin Nutr ESPEN	Spain	Not applicable (e.g. no	Review	Animal protein	Diabetes; Other: Kidney disease	Negative	Non-industry		No	No funding received			Negative	Negative/neutral	Non-industry	No	No	No	No	
Association between total, processed, red and white meat consumption and all-cause, CVD and IHD mortality: a meta-analysis of	I. Abete, D. Romaguera, A. R. Vieira, A. Lopez De Munain and T. Norat	2014	Nutrition	Br J Nutr	UK	Global	Meta analysis	Red meat; White	Cardiovascular disease; Ischemic Heart	Negative	Non-industry		No	No funding received			Negative	Negative/neutral	Non-industry	No	No	No	No	80
